# Acacetin Protects Against High Glucose-Induced Endothelial Cells Injury by Preserving Mitochondrial Function via Activating Sirt1/Sirt3/AMPK Signals

**DOI:** 10.3389/fphar.2020.607796

**Published:** 2020-12-18

**Authors:** Wei-Min Han, Xu-Chang Chen, Gui-Rong Li, Yan Wang

**Affiliations:** ^1^ Xiamen Cardiovascular Hospital, School of Medicine, Xiamen University, Xiamen, China; ^2^ Nanjing Amazigh Pharma Limited, Nanjing, China

**Keywords:** vascular endothelial cell injury, SIRT1, SIRT3, AMPK, atherosclerosis

## Abstract

The strategy of decreasing atherosclerotic cardiovascular disorder is imperative for reducing premature death and improving quality of life in patients with diabetes mellitus. The aim of this study was to investigate whether the natural flavone acacetin could protect against endothelial injury induced by high glucose and attenuate diabetes-accelerated atherosclerosis in streptozotocin-(STZ) induced diabetic ApoE^−/−^ mice model. It was found that in human umbilical vein endothelial cells (HUVECs) cultured with normal 5.5 mM or high 33 mM glucose, acacetin (0.3–3 μM) exerted strong cytoprotective effects by reversing high glucose-induced viability reduction and reducing apoptosis and excess production of intracellular reactive oxygen species (ROS) and malondialdehyde in a concentration-dependent manner. Acacetin countered high glucose-induced depolarization of mitochondrial membrane potential and reduction of ATP product and mitoBcl-2/mitoBax ratio. Silencing Sirt3 abolished the beneficial effects of acacetin. Further analysis revealed that these effects of acacetin rely on Sirt1 activation by increasing NAD^+^ followed by increasing Sirt3, pAMPK and PGC-1α. In STZ-diabetic mice, acacetin significantly upregulated the decreased signaling molecules (i.e. SOD, Bcl-2, PGC-1α, pAMPK, Sirt3 and Sirt1) in aorta tissue and attenuated atherosclerosis. These results indicate that vascular endothelial protection of acacetin by activating Sirt1/Sirt3/AMPK signals is likely involved in alleviating diabetes-accelerated atherosclerosis by preserving mitochondrial function, which suggests that acacetin may be a drug candidate for treating cardiovascular disorder in patients with diabetes.

## Introduction

Since atherosclerotic cardiovascular disease is the leading cause of increased mortality and a major cause of morbidity in patients with Type 1 and Type 2 diabetes worldwide (Beckman et al., 2002; Nathan, 2005; Orchard et al., 2006), decreasing atherosclerotic cardiovascular disorder is imperative to prevent premature death and improve quality of life in patients with diabetes mellitus. Sustained hyperglycemia (i.e. elevated blood glucose level) leads to macro- and microvascular complications; experimental and clinical studies have shown that hyperglycemia accelerated the formation of atherosclerosis in patients with diabetes (Kanter et al., 2007; Juutilainen et al., 2008; Beckman et al., 2013; Nagareddy et al., 2013; Paneni et al., 2013). Atherosclerosis results from diabetic macroangiopathy and is characterized by endothelial injury and dysfunction followed by formation of new intra-plaque vessel due to excessive/abnormal neovasculogenesis and angiogenesis, increased vascular permeability of the capillary vessels and tissue edema followed by atherosclerotic plaque hemorrhage and plaque rupture (Madonna et al., 2018).

Although the details of the mechanisms responsible for the accelerated formation of atherosclerosis lesion observed in diabetes are not fully understood (Calkin and Allen, 2006; Gray et al., 2013; Bornfeldt, 2014), it is generally recognized that mitochondrial dysfunction and increased ROS production are involved in endothelial impairment and acceleration of atherosclerosis in diabetes (Giacco and Brownlee, 2010; Rao et al., 2011; Yuan et al., 2019). The therapeutic strategies to treat diabetic atherosclerosis include approaches to prevent, inhibit or reverse diabetic cardiovascular complications. Previous studies have demonstrated that endothelial protection using exogenous antioxidants to modulate the endothelium-dependent vasodilation responses, the homeostatic endothelium-leukocyte interactions, the balance between pro- and anti-thrombotic properties and/or the vascular apoptotic responses is effective in attenuating vascular disorders in diabetes mellitus (Pratico, 2005; Taile et al., 2020). Activation of eNOS is atheroprotective in diabetes (Sharma et al., 2015). Enhancing mitochondrial biogenesis and reducing vascular mitochondrial ROS have emerged as crucial therapeutic approaches to ameliorate diabetic atherosclerosis injury (Wang et al., 2017).

We have previously found that the natural flavone acacetin (5,7-dihydroxy-4′-methoxyflavone), in addition to its atrial-selective anti-atrial fibrillation property (Li et al., 2008; Liu et al., 2016a), is cardioprotective against ischemia/reperfusion or hypoxia/reoxygenation injury and doxorubicin cardiotoxicity by its anti-oxidation, anti-inflammation, and anti-apoptosis properties (Liu et al., 2016b; Wu et al., 2018; Wu et al., 2020). Studies from other research teams have demonstrated that acacetin has anticancer (Fong et al., 2010; Kim et al., 2014), anti-peroxidation and anti-neuronal inflammation (Yin et al., 2008; Bu et al., 2019) properties. In addition, acacetin may reduce E-selectin expression in endothelial cells by regulating MAP kinase (Tanigawa et al., 2013). The present study was designed to investigate whether acacetin is protective against vascular endothelial injury in cultured human umbilical vein endothelial cells (HUVECs) exposed to high glucose conditions and against diabetes-accelerated atherosclerosis in diabetic ApoE^−/−^ mice induced by streptozotocin (STZ). Our results suggested that acacetin reduced vascular endothelial injury induced by high glucose and attenuated diabetes-accelerated atherosclerosis by Sirt1-mediated activation of Sirt3/AMPK signals.

## Materials and Methods

### Reagents and Antibodies

Acacetin and acacetin prodrug used in the present study were synthesized as described previously (Li et al., 2008; Liu et al., 2016a). Specific siRNA duplexes: control siRNA (sc-37007), Sirt1 siRNA (sc-40986), Sirt3 siRNA (sc-61555), and anti-β-actin antibody (sc-47778) were from Santa Cruz Biotechnology (Dallas, TX, United States). The primary antibodies anti-pAMPK (#2535) and anti-AMPK (#2532) antibodies were from Cell Signaling (Danvers, MA, United States); anti-Sirt1 (ab32441), anti-Sirt3 (ab217319), anti-Bcl-2 (ab182858), anti-Bax (ab32503), anti-PGC-1α (ab54481), anti-SOD1 (ab16831), anti-SOD2 (ab16956), anti-CD31 (ab9498) antibodies were from Abcam (Cambridge, MA, United States). Fluorescently–conjugated secondary antibodies (Alexa Fluor® 488, #A-11001; Alexa Fluor® 568, #A-11004; Alexa Fluor® 633, #A-21052) and Lipofectamine RNAiMAX Reagent were from Thermo Fisher Scientific (Waltham, MA, United States). A list of antibodies is shown in Supplementary Table S1.

### Cell Culture

Human umbilical vein endothelial cells (HUVECs) were obtained from ScienCell Research Laboratories (Carlsbad, CA, United States) and cultured in plates pre-coated with 0.2% gelatin in endothelial cell medium (ECM) supplemented with 5% fetal bovine serum, 1% penicillin/streptomycin with 1% endothelial cell growth supplement (ScienCell) at 37 °C, 5% CO_2_. For viability assay, cells were seeded in 96-well plates and grew to 70–80% confluence, then exposed to normal (5.5 mM) or high (33 mM) glucose (Sigma–Aldrich) medium for 5 days in the absence or presence of acacetin (0.3–3 μM). Cell viability was determined by 3-(4,5-dimethyl-2-thiazolyl)-2,5-diphenyl-2-H-tetrazolium bromide (MTT) (Solarbio Technology, Beijing, China) assay as described previously (Wu et al., 2018). Briefly, the cells were incubated with 0.5 mg/ml MTT for 4 h, and re-suspended in 150 μL of dimethyl sulfoxide (DMSO). Absorbance was measured at 575 nm using an Infinite M200 Pro NanoQuant (TECAN, Switzerland). The cells treated with DMSO were considered to be 100% viable.

In the experiments of HUVECs with inhibition of AMPK or nicotinamide phosphoribosyltransferase (NAMPT), the AMPK inhibitor dorsomorphin (Compound C, 10 μM) (Wu et al., 2018) was administered for 6 h before high glucose exposure to inhibit pAMPK, and the NAMPT inhibitor GMX-1778 (CHS-828, 10 nM) (Cerna et al., 2012) was administered for 24 h to inhibit NAD^+^ production before high glucose exposure. The concentrations used were based on previous reports (Cerna et al., 2012; Wu et al., 2018).

### Immunofluorescence Analysis

Immunofluorescence analysis was used to identify Sirt3 expression levels in aortic root sections and cultured HUVECs with different treatment. Briefly, aortic root sections were stained with anti-Sirt3 and anti-CD31 antibody. Following an overnight incubation with primary antibodies, aortic root sections or cells were washed three times with phosphate buffered saline (PBS), followed by a 1-h incubation with Alexa Fluor–conjugated secondary antibodies (Alexa Fluor® 488, A-11001 or A-11034; Alexa Fluor® 568, A-11004 or A-11011; Alexa Fluor® 633, A-21052 or A-21071) (Thermo Fisher Scientific) at room temperature, then mounted on DAPI-containing mounting media (Solarbio Technology, Beijing, China).

HUVECs were plated on confocal culture dish and cultured, thereafter labeled with MitoTracker® Red CMXRos (1:5,000) incubated at 37 °C for 20 min, then washed with PBS three times and fixed in 4% paraformaldehyde (P6148, Sigma-Aldrich, St. Louis, MO, United States) for 10 min. After permeabilization with 0.5% Triton X-100 (X100, Sigma-Aldrich) for 10 min, the cells were blocked with 10% goat serum (Solarbio Technology, Beijing, China) at 37 °C for 1 h, then incubated with anti-Sirt3 antibody (Solarbio Technology) at 4 °C overnight. Images were captured with a laser scanning confocal microscope (Olympus FV3000, Waltham, MA, United States).

### Flow Cytometry Analysis

Flow cytometry analysis was employed to assay the viability, apoptosis, ROS production and mitochondrial transmembrane potential in HUVECs using a flow cytometer (Beckman Coulter, United States) Flow cytometry analysis was employed to assay the viability, apoptosis, ROS production and mitochondrial transmembrane potential in HUVECs using a flow cytometer (Beckman Coulter). For viability and apoptosis determination, annexin V and propidium iodide staining were performed with the Annexin V-FITC apoptosis detection kit (Dojindo Molecular Technologies, Rockville, MD, United States) following the manufacturer’s instruction, and the stained cells were subjected to flow cytometry within 1 h.

For intracellular ROS analysis in HUVECs, 80-90% confluent cells seeded in six-well plate were pretreated with 0.3, 1, or 3 μM acacetin for 3 h, then exposed to 33 mM glucose medium with 0.3, 1, or 3 μM acacetin for 30 min, Afterward, the cell-permeable ROS probe 2′,7′-dichlorofluorescin diacetate (DCFH-DA, 10 μM) (Thermo Fisher Scientific) was applied and the cells were incubated for additional 30 min. After detachment with trypsin solution, the cells were washed with cool PBS for three times. Fluorescence intensity of DCFH-DA was determined in the single cell suspension using a flow cytometer (Beckman Coulter) with excitation wavelength of 488 nm and emission wavelength of 525 nm.

For the determination of mitochondrial transmembrane potential, HUVECs exposed to 33 mM glucose 5 days in the absence and presence of 0.3, 1 or 3 μM acacetin were incubated with JC-1 (2 μM), a membrane permeable dye for probing mitochondrial membrane potential (Solarbio Technology, Beijing, China), at 37 °C for 30 min. Green fluorescence reflected the monomeric form of JC-1, and red fluorescence reflected the aggregate form. Mitochondrial membrane potential was then analyzed with a flow cytometer.

### Mitochondrial Oxidative Stress and Functional Evaluation

HUVECs were cultured with 5.5 mM or 33 mM glucose (5 days) in the absence and presence of 0.3, 1, or 3 acacetin. The mitochondrial oxidative stress proteins (i.e. SOD activity and MDA content) of the harvested cells were measured using commercial Superoxide dismutase (SOD) Activity Colorimetric Assay Kit and kits and Lipid Peroxidation (MDA) Assay Kit (Jiancheng Institute of Bioengineering, Nanjing, China) as described previously (Yang et al., 2013). The intracellular ATP level was determined using ATP Bioluminescence Assay Kit (Beyotime Technology, Shanghai, China) following manufacture’s instruction.

### NAD^+^/NADH Determination

HUVECs were vulture with 33 mM glucose for 5 days in the absence or presence of 3 μM acacetin or the NAMPT inhibitor GMX-1778 (10 nM). The harvested cells were rinsed with PBS twice and centrifuged 4 × at 1,000 rpm for 10 min, then oxidized nicotinamide adenine dinucleotide (NAD^+^) and reduced nicotinamide adenine dinucleotide hydride (NADH, a reduced form of nicotinamide adenine dinucleotide) levels were quantified using an EnzyChrom™ NAD^+^/NADH assay kit (Bioassay Systems, Hayward, CA, United States) following the manufacturer’s instruction.

### Small Interfering RNA (siRNA)

The siRNA technique was used to silence specific genes in HUVECs. The cells at 40%-50% confluence were transfected with specific siRNA duplexes (60 nM, Santa Cruz Biotechnology) using Lipofectamine RNAiMAX Reagent (Thermo Fisher Scientific) following the manufacturer’s instruction. After 48 h transfection of control siRNA, Sirt1 siRNA or Sirt3 siRNA (Santa Cruz Biotechnology), the cells were incubated with 5.5 mM or 33 mM glucose culture medium in the absence or presence of 3 μM acacetin for 5 days, then collected for western blot analysis. The silencing efficiency of Sirt1 and Sirt3 proteins was significant, which is shown in Supplementary Figure S1.

### Western Blot Analysis

Western blot analysis was employed to determine the expression of specific proteins in aortic tissues and cultured HUVECs. Proteins of aortic tissue homogenate lysates or HUVECs lysates prepared in sodium dodecyl sulfate (SDS) lysis buffer were extracted with RIPA buffer supplemented with protease and phosphatase inhibitors on ice, and protein concentration was determined using the BCA protein assay kit (Solarbio, Beijing, China) as described previously (Liu et al., 2016b; Wu et al., 2018). The proteins of HUVEC mitochondria were isolated using the Mitochondria Isolation Kit for Cultured Cells (Thermo Fisher Scientific) following the manufacturer’s instruction. SDS-PAGE and polyvinylidene difluoride (PVDF) membranes (Bio-Rad, Hercules, CA, United States) were applied to separate the proteins samples. The membranes were blocked with 5% fat-free dry milk in 0.1% Tris-buffered saline with Tween for 2 h and then probed with primary antibodies (1:1,000) overnight at 4 °C overnight. After washout, membranes were incubated with secondary antibody (1:10,000) for 1 h at room temperature. Blots were visualized with ECL™ reagents (Advansta, Menlo Park, CA, United States), and the protein signals were captured with FluorChem E chemiluminescence detection system (ProteinSimple, San Jose, CA, United States). All cellular western blots were repeated at least five times, and the signal intensity of the immunoreactive bands was quantified using Image J software (NIH, Bethesda, MD, United States) and normalized to that of β-actin in each sample.

### Animal Experiments

The animal experimental protocol was approved by the Animal Care and Ethics Committee of Xiamen University. Male ApoE^−/−^ mice were obtained from Beijing Vital River Laboratory Animal Technology (Beijing, China) and raised in Laboratory Animal Center of Xiamen University. The age-matched animals were cared for following the Guide for Care and Use of Laboratory Animals published by National Institutes of Health (NIH Publication No. 85-23, revised 1996) of the United States. Type 1 diabetic mouse model was established in 7-week-old ApoE^−/−^ mice by intraperitoneal injection of STZ (daily 50 mg/kg, Sigma-Aldrich, MO, United States) or vehicle citric acid (control) for five consecutive days. Random blood glucose was measured 2 weeks after last streptozotocin injection with the Accu-Chek Performa glucometer (Roche, United States), and only animals with blood glucose >16.7 mM were classified as diabetic. Experiments were assigned as control, control with acacetin treatment, STZ-diabetes, STZ-diabetes with acacetin treatment. Animals with acacetin treatment received acacetin prodrug subcutaneously at 20 mg/kg twice daily (the prodrug can be metabolized into acacetin) (Liu et al., 2016a; Liu et al., 2016b), and control animals received subcutaneous equivolume vehicle (0.9% saline). All animals were maintained at room temperature (23 ± 2 °C) with a 12 h light/dark cycle and free access to basic diet and water for additional 12 weeks. Bodyweight and blood glucose were measured every 4 weeks. When the animals were sacrificed at the end of experiments, the blood was collected in a centrifuged tube with 25 µL of heparin. After centrifugation, plasma was collected to analyze the amount of cholesterol and triglycerides and high-density lipoprotein, low-density lipoprotein, lipoprotein A, lipoprotein B with Infinity reagent (Thermo Fisher Scientific). Aortas were isolated for immunohistochemistry, immunofluorescence, and western blot analyses.

### Atherosclerotic Assay

Aortic root lesion and en face lesion areas of whole aorta were fixed with 4% paraformaldehyde and stained with Oil Red O as described previously (Dong et al., 2010). Aortic root sections (10 μm thickness) of 4% paraformaldehyde-fixed, OCT-embedded frozen hearts were cut from the aortic valve leaflets at 150–200 μm following the valve leaflet. Sections were concurrently stained with 0.5% w/v Oil Red O and hematoxylin and eosin (HE) to assess atherosclerotic lesions as described previously (Kennedy et al., 2009). The images were captured with an Olympus BX40 microscope (×20 magnification). All image quantifications were analyzed as described previously (Ganguly et al., 2017).

### Statistical Analysis

Statistical analyses were performed with GraphPad Prism 6.0 (GraphPad Software, Inc., San Diego, CA, United States). Results are presented as means ± SEM. One-way ANOVA followed by Bonferroni post hoc test were used for comparison among groups. *p* values <0.05 were considered statistically significant.

## Results

### Acacetin Prevents High Glucose Exposure-induced Viability Reduction and Increase in Apoptosis and Oxidative Stress in Human Umbilical Vein Endothelial Cells

**FIGURE 1:**
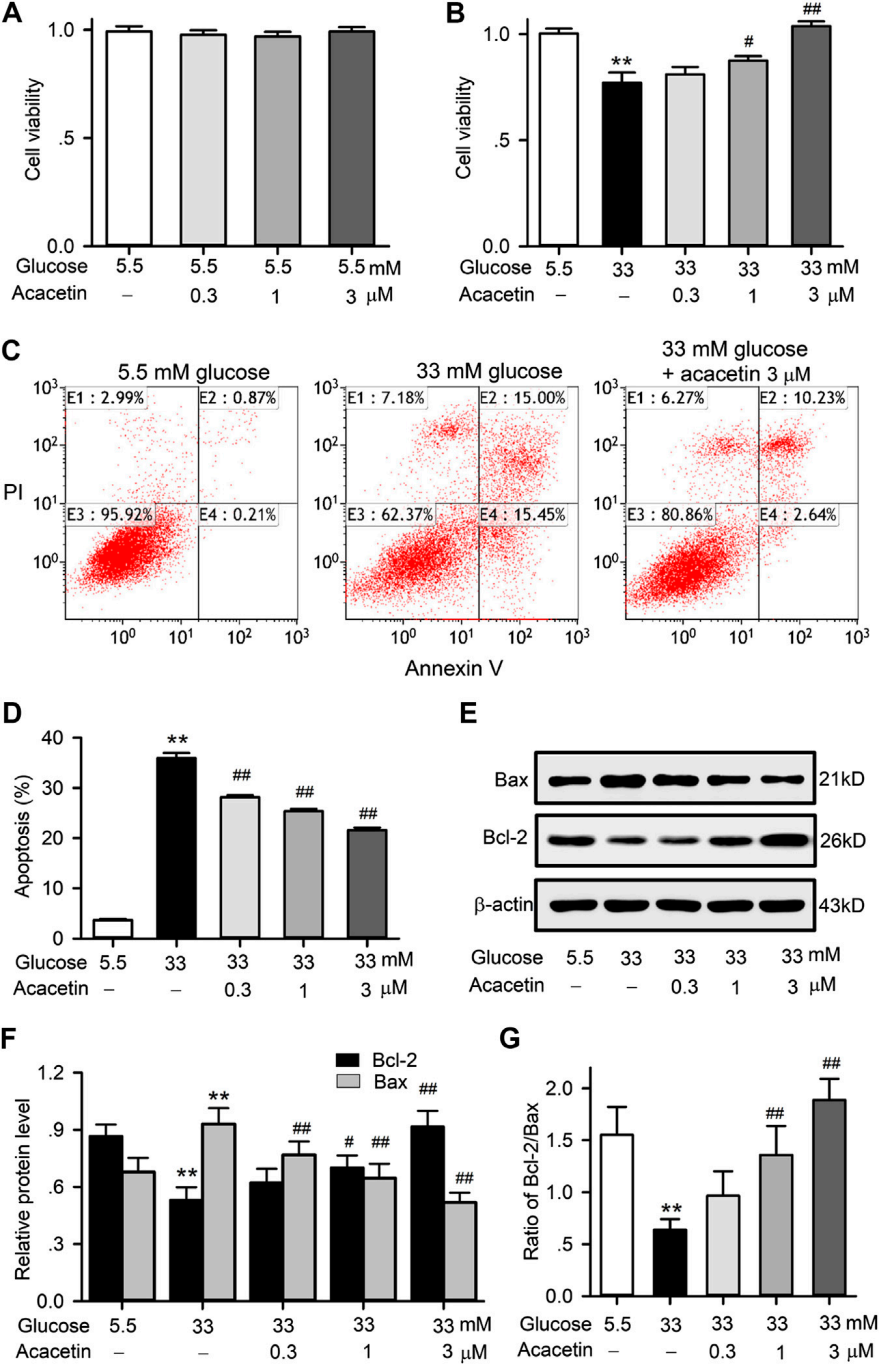
Effects of acacetin on cell viability and apoptosis induced by high glucose exposure in HUVECs. **(A)** Acacetin had no effect on cell viability in HUVECs cultured with 5.5 mM glucose medium. **(B)** Acacetin countered viability reduction induced by high glucose medium culture in a concentration-dependent manner. **(C)** Flow cytometry graphs for cell apoptosis (E2 + E4) in cells cultured with 5.5 mM or 33 mM glucose medium, or 33 mM glucose plus 3 μM acacetin. **(D)** Acacetin decreased apoptosis induced by high glucose culture in a concentration-dependent manner. **(E)** Western blots of apoptosis-related proteins Bax and Bcl-2 in HUVECs culture with 5.5 mM glucose or 33 mM high glucose, or 33 mM glucose with 0.3, 1 and 3 μM acacetin. **(F)** Acacetin reversed the Bax increase and Bcl-2 decrease induced by high glucose culture in a concentration-dependent manner. **(G)** Acacetin countered the decreased ratio of Bcl-2/Bax in a concentration-dependent manner. *n* = 5 individual experiments, **p < 0.01 vs. 5.5 mM glucose; ^#^ *p* < 0.05, ^##^ *p* < 0.01 vs. 33 mM glucose).

To determine the potential protection of acacetin against vascular endothelial cells injury by high glucose conditions, HUVECs were cultured with normal glucose (5.5 mM) medium or a high glucose (33 mM) medium in the absence or presence of acacetin (0.3–3 μM). Acacetin had no effect on viability of HUVECs cultured with normal glucose medium (Figure 1A), while it reversed high glucose-induced viability reduction in a concentration-dependent manner (Figure 1B). Flow cytometry analysis revealed that the viability reduction by high-glucose was related to increase in apoptosis (Figure 1C). Acacetin significantly reversed the increase in apoptosis (Figures 1C,D). Results of western blot analysis showed that pro-apoptotic protein Bax was increased, while anti-apoptotic protein Bcl-2 was decreased in HUVECs cultured with 33 mM glucose medium. Acacetin treatment reversed the Bax increase, enhanced the reduced Bcl-2, and increased the reduced Bcl-2/Bax ratio in a concentration dependent manner (Figures 1E–G). These results suggest that acacetin protects HUVECs against high glucose injury by inhibiting apoptosis.

**FIGURE 2:**
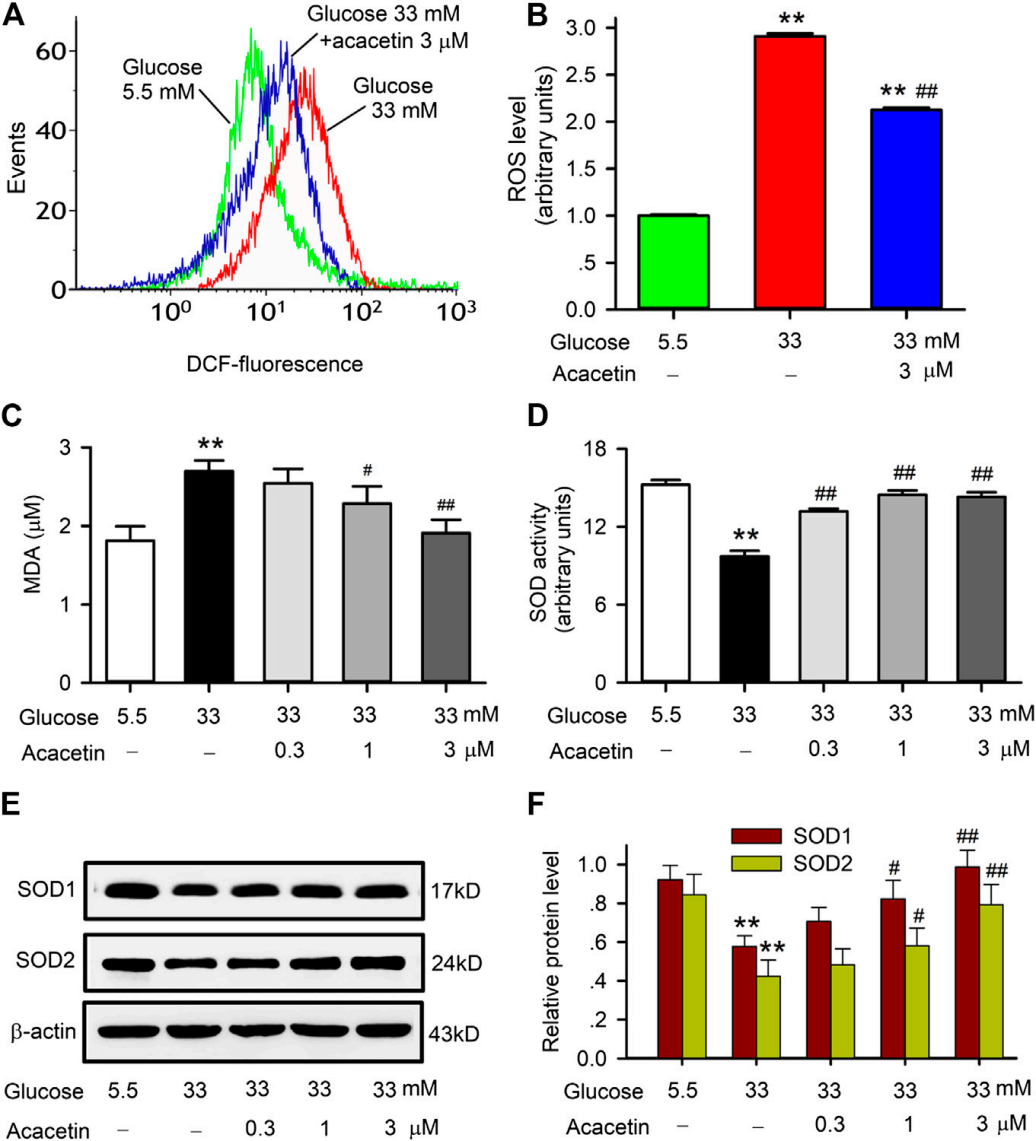
Effects of acacetin on ROS production and alteration of superoxide dismutase in HUVECs induced by high glucose exposure. **(A)** Flow cytometry graphs of ROS production in HUVECs cultured with 5.5 mM glucose medium, 33 mM glucose medium or 33 mM glucose medium plus 3 μM acacetin. **(B)** Acacetin decreased high glucose-induced ROS increase. **(C)** Acacetin decreased high glucose-induced MDA increase in a concentration-dependent manner. **(D)** Acacetin reversed high glucose-induced decrease in SOD activity in a concentration-dependent manner. **(E)** Western blots of SOD1 and SOD2 in HUVECs cultured with 5.5 mM glucose medium, 33 mM glucose medium or 33 mM glucose medium plus acacetin. **(F)** Acacetin reversed high glucose-induced reduction of SOD1 and SOD2 proteins in a concentration-dependent manner. *n* = 5 individual experiments, **p < 0.01 vs. 5.5 mM glucose; ^#^ *p* < 0.05, ^##^ *p* < 0.01 vs. 33 mM glucose).

It is generally believed that excess production of ROS is involved in endothelial apoptosis in diabetic cardiovascular complications. ROS production and the expression of antioxidant proteins SOD1 and SOD2 were therefore determined in HUVECs cultured with 33 mM glucose medium (Figure 2). High glucose induced an increase of ROS production in HUVECs, acacetin (3 μM) significantly impeded the ROS production (Figures 2A,B). Acacetin reversed the increase in malondialdehyde (MDA) content (Figure 2C) and reversed the reduction in SOD activity (Figure 2D) in HUVECs cultured with 33 mM glucose in a concentration-dependent manner. Moreover, high glucose-induced reductions of SOD1 and SOD2 proteins were also reversed in HUVECs treated with acacetin (Figures 2E,F).

### Acacetin Attenuates High Glucose-Induced Mitochondrial Injury in Human Umbilical Vein Endothelial Cells

**FIGURE 3:**
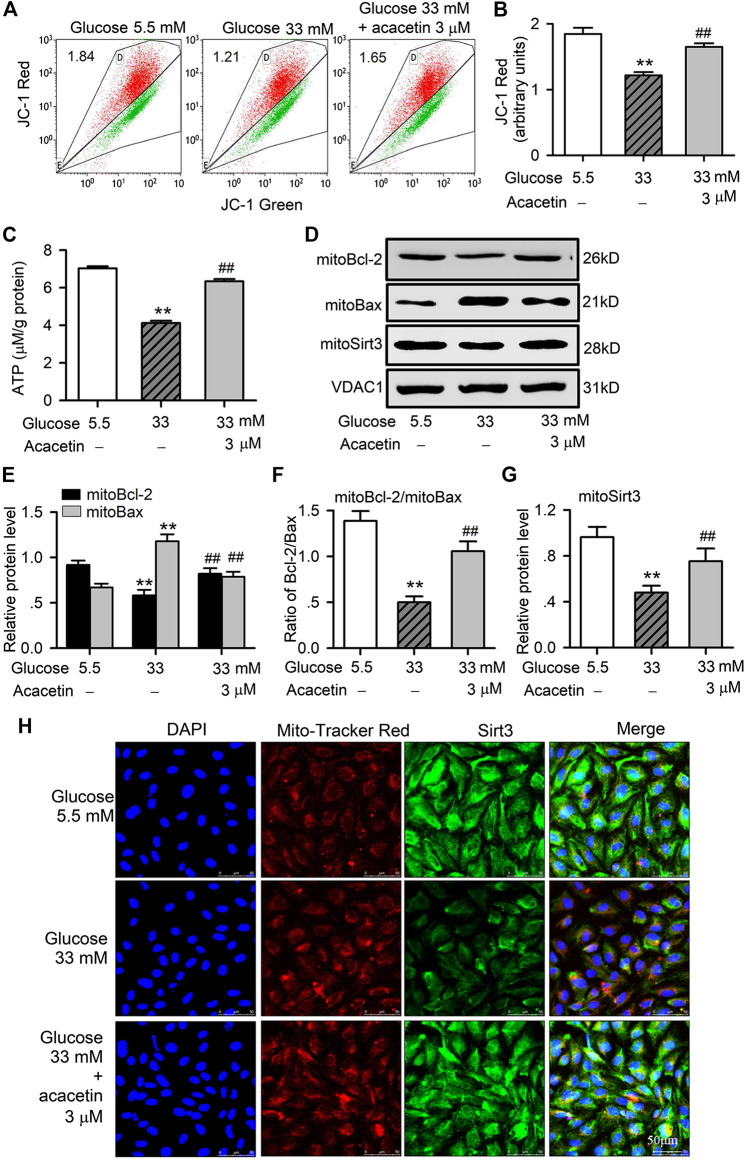
Protective effects of acacetin against mitochondrial dysfunction and injury induced by high glucose exposure in HUVECs. **(A)** Flow cytometry graphs for determining mitochondrial transmembrane potential in HUVECs cultured with 5.5 mM glucose medium, 33 mM glucose medium or 33 mM glucose medium plus 3 μM acacetin. **(B)** Acacetin reversed the decrease in mitochondrial transmembrane potential in cells with high glucose exposure. **(C)** Acacetin countered high glucose-induced decrease in ATP production. **(D)** Western blots of mitochondrial Bcl-2, Bax, and Sirt3 in cells cultured with 5.5 mM glucose medium, 33 mM glucose medium or 33 mM glucose medium plus 3 μM acacetin. **(E)** Relative (to VDAC1, voltage-dependent anion channel 1) mitochondrial Bcl-2 and Bax protein levels in cells cultured with 5.5 mM glucose medium, 33 mM glucose medium or 33 mM glucose medium plus 3 μM acacetin. **(F)** Acacetin reversed the reduced ratio of mitochondrial Bcl-2/Bax proteins. **(G)** Acacetin reversed the high glucose-induced reduction of Sirt3 protein. **(H)** Sirt3 immunocytochemical staining and mitoTracker Red co-staining (×80 magnification) of HUVECs cultured with 5.5 mM glucose medium, 33 mM glucose medium or 33 mM glucose medium plus 3 μM acacetin. *n* = 5 individual experiments, **p < 0.01 vs. 5.5 mM glucose; ^#^ *p* < 0.05, ^##^ *p* < 0.01 vs. 33 mM glucose.

To investigate whether the potential protection of acacetin against high glucose-induced endothelial injury is related to preserving mitochondrial function, we determined mitochondrial transmembrane potential, ATP production and mitochondrial Bax (mitoBax) and Bcl-2 (mitoBcl-2) in HUVECs (Figure 3). The HUVECs cultured with 33 mM glucose medium showed significant decrease in mitochondrial transmembrane potential (Figures 3A,B) and ATP production (Figure 3C), and these reductions were countered in cells treated with 3 μM acacetin (Figures 3A–C). Moreover, mitoBax was increased, while mitoBcl-2 and mitoSirt3 were reduced in HUVECs cultured with 33 mM glucose medium (*p* = 0.0038 vs. 5.5 mM glucose). Acacetin (3 μM) significantly countered the increase of mitoBax, the decrease of mitoBcl-2, mitoBcl-2/mitoBax ratio and mitoSirt3 induced by high glucose (*p* = 0.0051 vs. 33 mM glucose alone) (Figures 3D–G). Immunocytochemistry analysis also revealed that the high glucose-induced decrease of mitoSirt3 was reversed in cells treated with 3 μM acacetin (Figure 3H). These results further confirm that vascular endothelial protection of acacetin against high glucose injury is due to preserving mitochondrial function.

### Sirt3 Is Involved in the Protective Effects of Acacetin Against High Glucose-Induced Injury in Human Umbilical Vein Endothelial Cells

**FIGURE 4:**
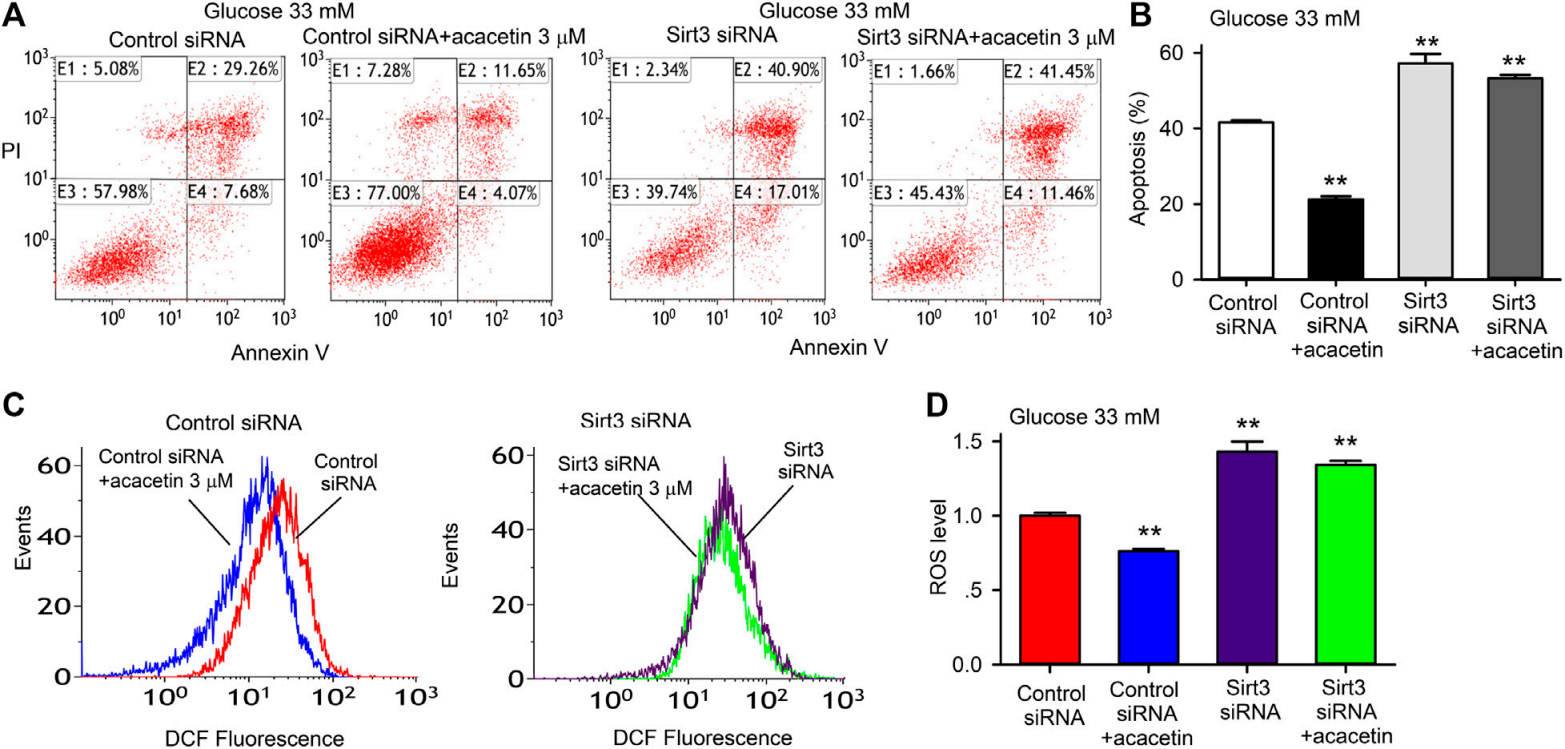
Sirt3 silencing and acacetin effects on apoptosis and ROS production induced by high glucose exposure in HUVECs. **(A)** Flow cytometry graphs for analyzing apoptosis (E2 + E4) in cells transfected with control siRNA or Sirt3 siRNA and cultured with 33 mM glucose medium in the absence or presence of 3 μM acacetin. **(B)** Acacetin decreased apoptosis in cells transfected with control siRNA, but not in cells transfected with Sirt3 siRNA. **(C)** Flow cytometry graphs for determining ROS levels in cells transfected with control siRNA or Sirt3 siRNA and cultured with 33 mM glucose medium in the absence or presence of 3 μM acacetin. **(D)** Acacetin reduced ROS production in cells transfected with control siRNA, but not in cells transfected with Sirt3 siRNA. *n* = 5 individual experiments, **p < 0.01 vs. control siRNA.

To determine the potential role of Sirt3 in acacetin protection against high glucose-induced endothelial injury, siRNA molecules targeting Sirt3 were employed in HUVECs. Figure 4 illustrates the effects of acacetin on high glucose-induced apoptosis and ROS production in HUVECs transfected with control siRNA or Sirt3 siRNA cultured with 33 mM glucose medium in the absence or presence of 3 μM acacetin. Acacetin significantly decreased high glucose-induced apoptosis (Figures 4A,B), ROS production (Figures 4C,D) in cells transfected with control siRNA, but not in cells transfected with Sirt3 siRNA. These results showed that silencing Sirt3 caused a further high glucose-induced increase in apoptosis and ROS production, and importantly abolished acacetin effects. These results indicate that Sirt3 plays an important role in vascular endothelial protection against high glucose-induced injury.

**FIGURE 5:**
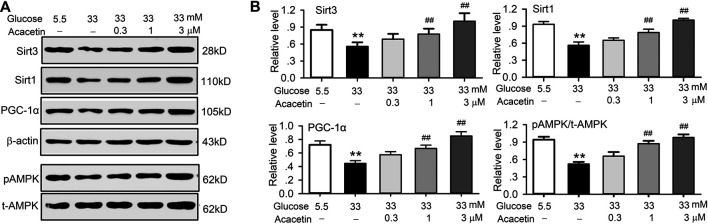
Acacetin rescued the impaired signal molecules induced by high glucose exposure in HUVECs. **(A)** Western blots of Sirt3 Sirt1, PGC-1α, pAMPK, t-AMPK in HUVECs cultured in 5.5 mM glucose or 33 mM glucose in the absence or presence of 0.3, 1, or 3 μM acacetin. **(B)** Acacetin reversed the decrease of Sirt3, Sirt1, PGC-1α and pAMPK in a concentration-dependent manner. *n* = 5 individual experiments, **p < 0.01 vs. 5.5 mM glucose; ^##^ *p* < 0.01 vs. 33 mM glucose alone.

Western blot analysis in HUVECs revealed that acacetin not only reversed the high glucose-induced decrease of Sirt3 expression in a concentration-dependent manner, but also the high glucose-induced downregulation of Sirt1, PGC-1α, and pAMPK expression (Figure 5). These results suggest that acacetin protection of vascular endothelial cells is due to the drug increasing Sirt1, Sirt3, pAMPK, and PGC-1α. Furthermore, AMPK signaling pathway is a fuel sensor and regulator that promotes ATP-producing and inhibits ATP-consuming pathways in various tissues (Ke et al., 2018). The increase of ATP levels in HUVECs by acacetin (Figure 3C) may be related to increasing AMPK phosphorylation.

### The Protective Effect of Acacetin Against Endothelial Cells Injury Involves Sirt1-Mediated Activation of Sirt3/AMPK Signals

**FIGURE 6:**
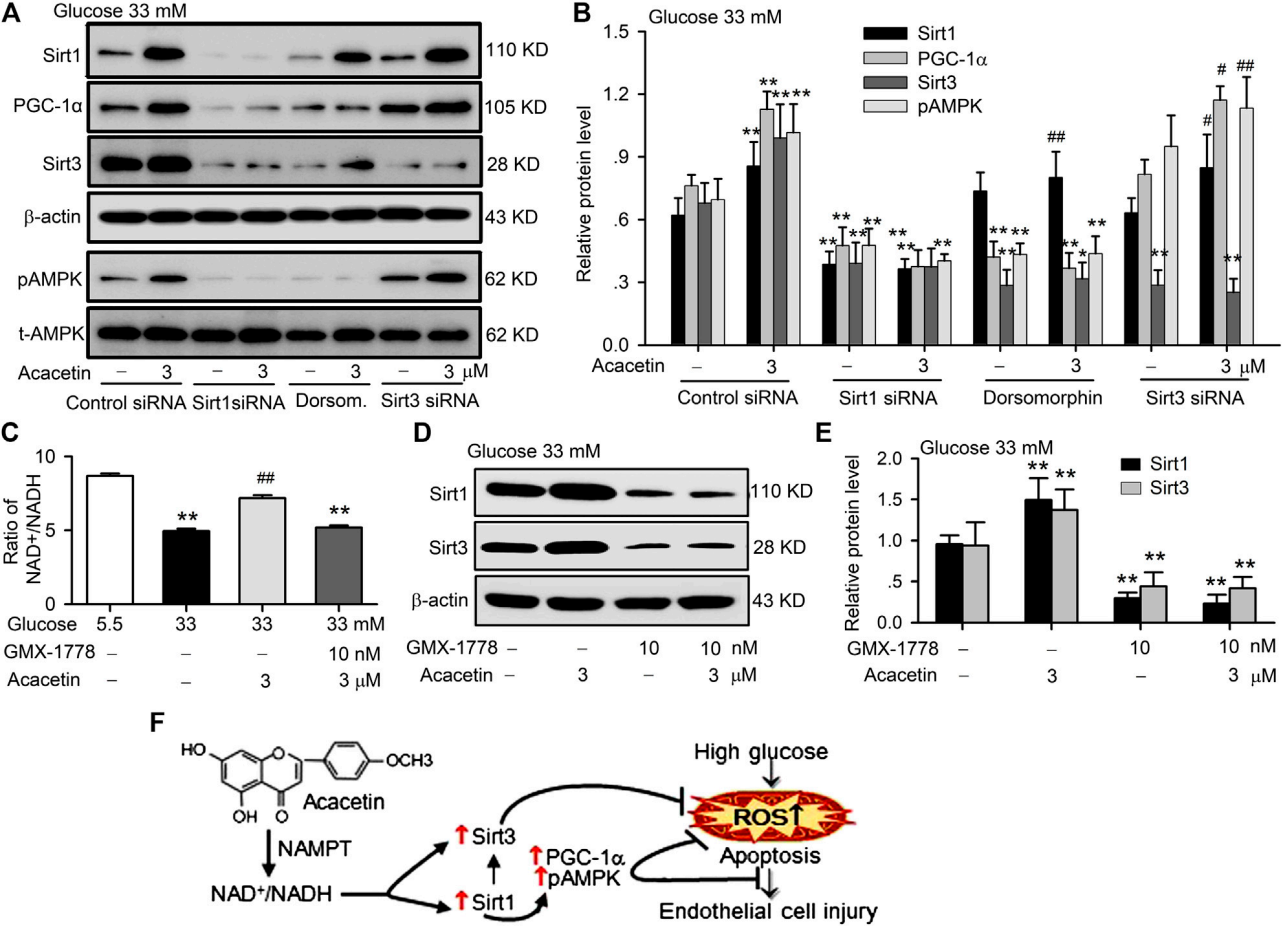
Sirt1 mediates the vascular protection of acacetin against hyperglycemia-induced injury by activating Sirt3/AMPK/PGC-1α. **(A)** Western blots of Sirt1, PGC-1α, Sirt3, pAMPK, t-AMPK in HUVECs cultured with 33 mM glucose and transfected with Sirt1 siRNA or Sirt3 siRNA or treated with the AMPK inhibitor dorsomorphin (Dorsom. 10 μM) in the absence or presence of 3 μM acacetin. **(B)** Silencing Sirt1, but not Sirt3 or inhibition of pAMPK decreased Sirt1 expression and abolished the Sirt1 activation by acacetin (*n* = 5 individual experiments, **p < 0.01 vs. control siRNA 33 mM glucose alone; ^#^ *p* < 0.05, ^##^ *p* < 0.01 vs. 33 mM glucose alone). **(C)** Acacetin reversal of the high glucose-induced reduction NAD^+^/NADH ratio was decreased by the NAMPT inhibitor GMX-1778 (10 nM) (*n* = 5 individual experiments, **p < 0.01 vs. 5.5 mM glucose alone; ^##^ *p* < 0.01 vs. 33 mM glucose alone). **(D)** Western blots of Sirt1 and Sirt3 in cells cultured with 33 mM glucose medium in the absence or presence of 3 μM acacetin and 33 mM glucose medium with 10 nM GMX-1778 in the absence or presence of 3 μM acacetin. **(E)** GMX-1778 decreased the expression of Sirt1 and Sirt3 proteins and abolished the upregulation of Sirt1 and Sirt3 by acacetin (*n* = 5 individual experiments, **p < 0.01 vs. 33 mM glucose alone). **(F)** Schematic graph shows that acacetin protects against high glucose-induced vascular endothelial cell injury by increasing NAD^+^/NADH followed by Sirt1-mediated activation of Sirt3/AMPK/PGC-1α, thereby elevating mitochondrial antioxidation and inhibiting apoptosis.

To further identify the molecular target of acacetin for the protection against high glucose- or hyperglycemia-induced vascular endothelial injury, siRNA molecules targeting Sirt1 or Sirt3 and the AMPK inhibitor dorsomorphin were utilized in HUVECs cultured with 33 mM glucose to determine their effects on acacetin-induced upregulation of Sirt1, PGC-1α, Sirt3, and pAMPK (Figures 6A,B). It is interesting to note that silencing Sirt1 abolished the acacetin-induced increase of Sirt1, PGC-1α, Sirt3, and pAMPK, while silencing Sirt3 only inhibited the upregulation of Sirt3, but not the acacetin-induced increase of Sirt1, pAMPK or PGC-1α. The AMPK inhibitor dorsomorphin decreased the expression of pAMPK, PGC-1α, and Sirt3; dorsomorphin fully abolished the increase of pAMPK and PGC-1α by acacetin, and greatly reduced the Sirt3 but not Sirt1 increase by acacetin. These results indicate that protective effect of acacetin against high glucose-induced vascular endothelial injury is related to reversing the high glucose- or hyperglycemia-induced reductions of pAMPK, Sirt3 and PGC-1α molecules by activating Sirt1.

It is well recognized that sirtuins are NAD^+^- dependent deacetylases that controls metabolism, and biosynthesis of NAD^+^ is mediated by NAMPT (Imai and Guarente, 2016). The sirtuins activity is correlated to ratio of NAD^+^/NADH (Anderson et al., 2017). We therefore determined the potential effects of acacetin on the NAD^+^/NADH ratio and expression of Sirt1 and Sirt3 in the absence or presence of the NAMPT inhibitor GMX-1778 (CHS-828) (Cerna et al., 2012). The NAD^+^/NADH ratio was reduced in HUVECs cultured with 33 mM glucose medium, and the reduction was countered in cells treated with 3 μM acacetin, but not in cells co-treated with 10 nM GMX-1778 (Figure 6C). Moreover, GMX-1778 not only induced a further decrease of Sirt1 and Sirt3 proteins, but also prevented the acacetin-induced recovery of Sirt1 and Sirt3 proteins (Figures 6D,E) in HUVECs cultured with 33 mM glucose medium. These results indicate that the acacetin protection against high glucose-induced vascular endothelial injury is related to increasing NAD^+^/NADH followed by Sirt1-mediated activation of Sirt3/AMPK/PGC-1α signals, thereby elevating mitochondrial antioxidation and inhibiting apoptosis (Figure 6F).

### Acacetin Improves the Alterations of Lipid Profiles in STZ-Diabetic ApoE^−/−^ Mice

The potential protection of acacetin against vascular injury induced by hyperglycemia was confirmed in an ApoE^−/−^ mouse model of STZ-diabetes. The lipid profiles of this diabetic model were illustrated in Supplementary Table S2. Triglyceride, total cholesterol, low-density lipoprotein, lipoprotein A, and lipoprotein B were increased in STZ-diabetic mice by 104.6%, 52.7%, 610%, 398.8% and 120.1% respectively (*n* = 8, *p* = 0.0021–0.00016 vs. control), while high-density lipoprotein was decreased by 33.1% (*n* = 8, *p* = 0.0011 vs. control) in STZ-diabetic ApoE^−/−^ mice. Acacetin treatment did not alter the basal lipid profiles in control animals (Supplementary Table S2); however, it reduced the percent increases of triglyceride, total cholesterol, low-density lipoprotein, lipoprotein A, and lipoprotein B to 37.9%, 11.6%, 387.1%, 98.1% and 11.4% (*n* = 8, *p* = 0.015–0.00018 vs. STZ-diabetes), and the percent decrease of high-density lipoprotein to 10.4% (*p* = 0.0043 vs. STZ-diabetes). However, the random blood glucose level was not significantly reduced in STZ-diabetic ApoE^−/−^ mice treated with acacetin (23.1 ± 2.4 mM vs. 20.4 ± 2.1 mM of STZ-diabetes, *p* = 0.251) and final bodyweight showed no differences between STZ-diabetic animals (25.91 ± 1.24 g) and STZ-diabetic animals treated with acacetin (27.87 ± 1.47 g) (Supplementary Table S2). These results suggest that acacetin may improve the alterations in lipid profiles without reducing blood glucose level in STZ-diabetic ApoE^−/−^ mice.

### Acacetin Attenuates Diabetes-Accelerated Atherosclerosis in STZ-Diabetic ApoE^−/−^ Mice

**FIGURE 7:**
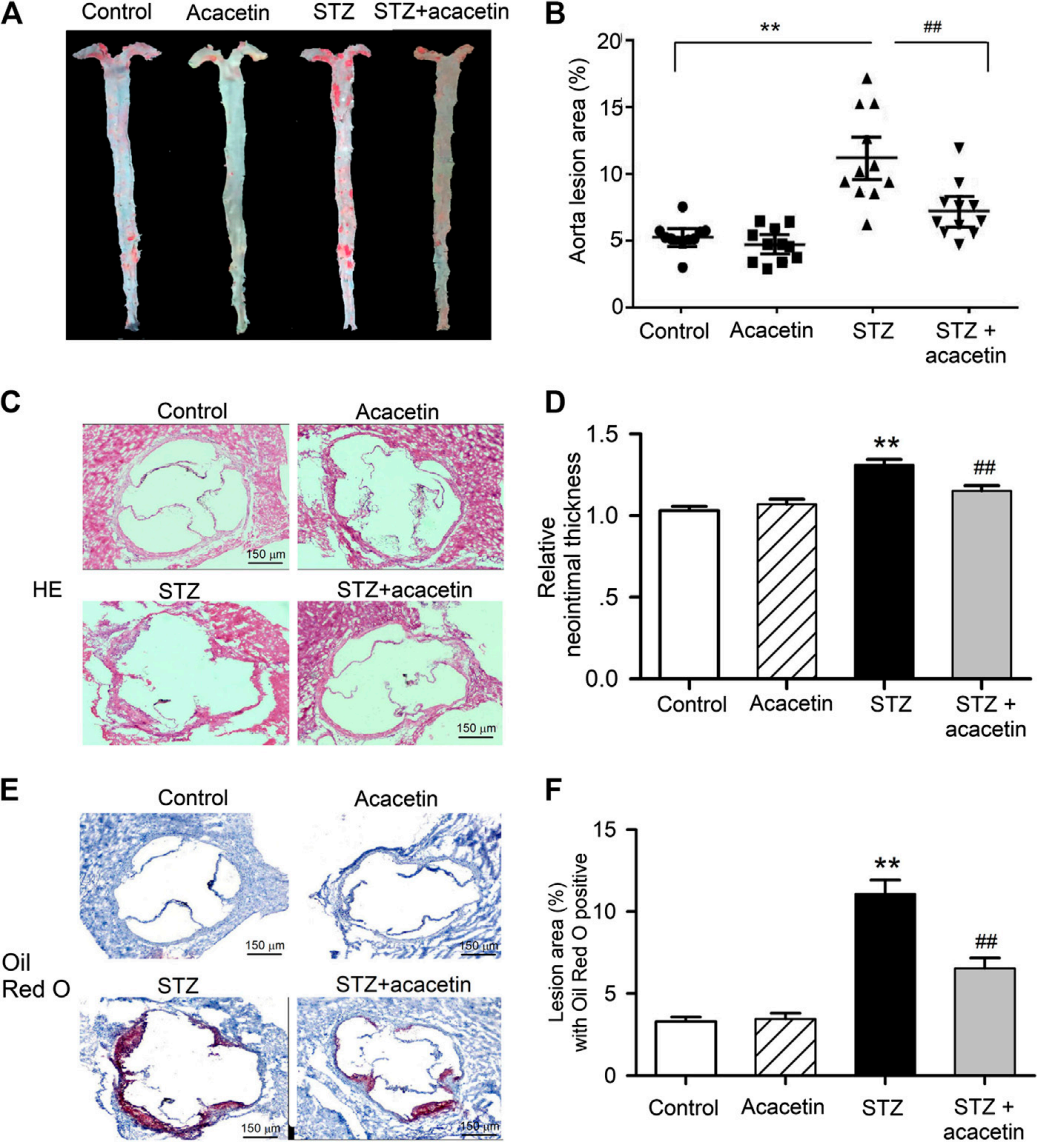
Acacetin attenuates STZ-induced atherosclerosis in STZ-diabetic ApoE^−/−^ mice. **(A)** Oil Red O staining of mouse aortas in control, with acacetin treatment, STZ, and STZ with acacetin treatment. **(B)** Analysis of aorta lesion areas stained with Oil Red O in A. **(C)** HE staining of aortic root section from mice in control, with acacetin, STZ, and STZ with acacetin treatment. **(D)** Analysis of neointimal thickness in C. **(E)** Oil Red O staining of the same aortic root sections from mice as in C. **(F)** Lesion area of aortic root section with Oil Red O positive in E (×4 magnification). n = 11 **p < 0.01 vs. control or acacetin; ^##^ *p* < 0.01 vs. STZ.

The Oil Red O staining of en face aorta showed that atherosclerotic lesion was greater in STZ-diabetic ApoE^−/−^ mice than nondiabetic ApoE^−/−^ mice (control), and the lesion was reduced in STZ-diabetic ApoE^−/−^ mice treated with acacetin (Figure 7A). The analysis of Oil Red O stained area (Figure 7B) showed that acacetin treatment significantly decreased the aorta lesion area from 11.0 ± 1.12% (*n* = 11, *p* = 0.0014 vs. control) in non-treated STZ-diabetic ApoE^−/−^ mice to 7.0 ± 0.6% (*p* = 0.0071 vs. STZ-diabetes).

The aortic root sections were stained with hematoxylin and eosin (Figure 7C) or Oil Red O (Figure 7E) to show the neointimal thickness and the lesion burden, in aortic roots of animals with different treatment. The increases in neointimal thickness (Figure 7D) and lesion burden (Figure 7F) were significantly reduced in STZ-diabetic ApoE^−/−^ mice treated with acacetin (*n* = 8, *p* = 0.0071 vs. STZ-diabetes). These results suggest that acacetin provides effective vascular protection against hyperglycemia-induced injury in STZ-diabetic ApoE^−/−^ mice.

### Effects of Acacetin on Protective Kinase Proteins in Aortic Tissues of STZ-Diabetic ApoE^−/−^ Mice

**FIGURE 8:**
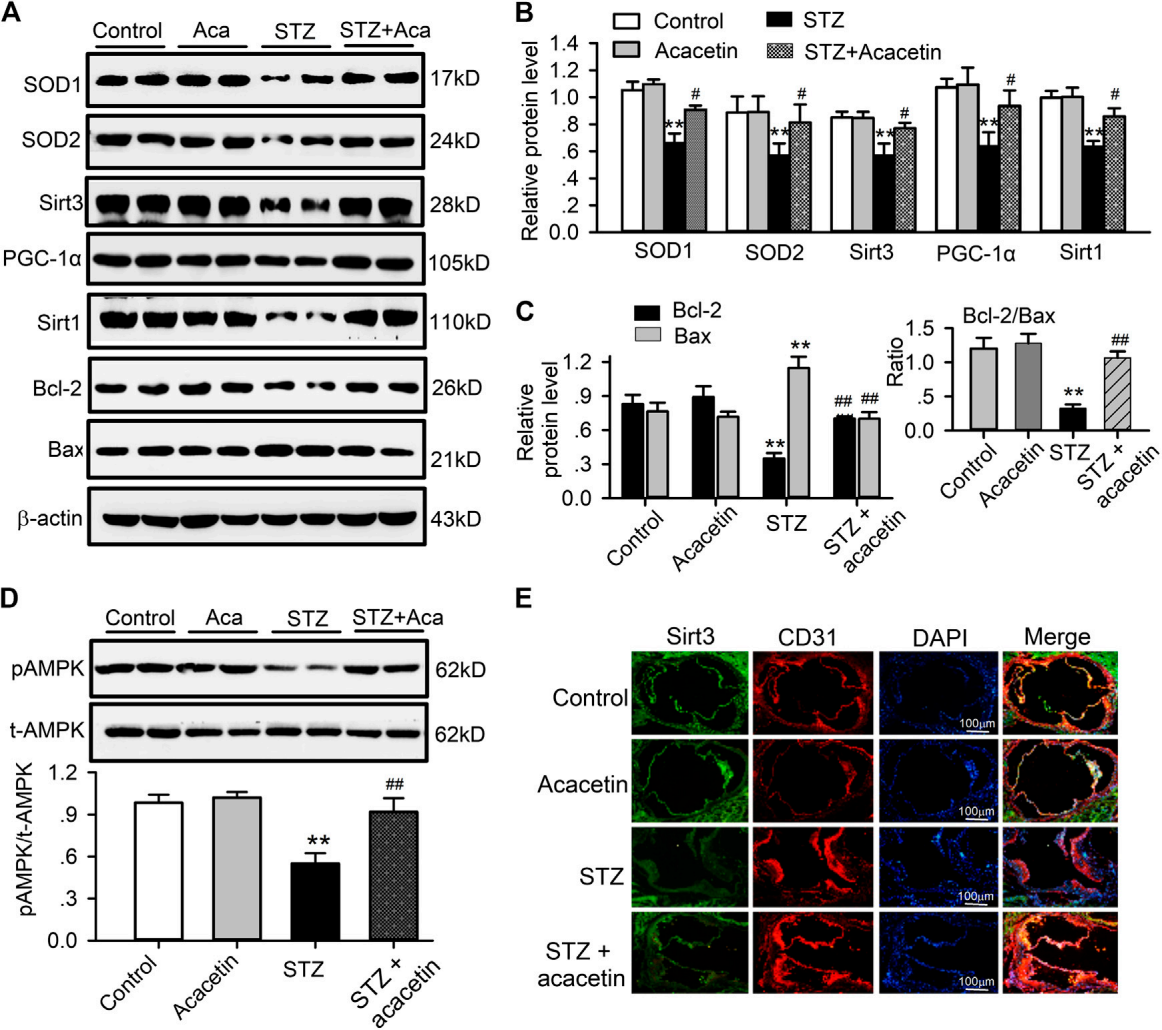
Alterations of related proteins in aortic tissues of STZ-diabetic ApoE^−/−^ mice. **(A)** Western blots of SOD1, SOD2, Sirt3, PGC-1α, Sirt1, Bcl-2, Bax, and β-actin in control aorta with and without acacetin and in STZ-diabetic ApoE^−/−^ mice aorta with and without acacetin. **(B)** Analysis of SOD1, SOD2, Sirt3, PGC-1α, and Sirt1 (relative to β-actin). **(C)** Analysis of Bcl-2 and Bax proteins, relative to β-actin. Inset: ratio of Bcl-2/Bax. **(D)** Western blots of pAMPK and t-AMPK (total AMPK) and relative level of pAMPK/t-AMPK. *n* = 6, *p < 0.05, **p < 0.01 vs. control or acacetin; ^#^ *p* < 0.05, ^##^ *p* < 0.01 vs. STZ. **(E)** Sirt3 and CD31 immunohistochemical staining of aortic root section from mice of control, with acacetin, STZ, and STZ with acacetin treatment (×10 magnification).

It is well documented that diabetic atherosclerosis is associated with downregulation of a series of signaling molecules involved in energy metabolism, anti-oxidation and anti-apoptosis, e.g. SOD1, SOD2, Sirt1, Sirt3, AMPK, PGC-1α, and Bcl-2 (Low Wang et al., 2016; Shah and Brownlee, 2016). To investigate whether the vascular protection of acacetin is mediated by upregulating these signaling molecules, we determined the expression of SOD1, SOD2, Sirt1, pAMPK, PGC-1α, Bcl-2, and Sirt3 in aortic tissues in non-diabetic mice, STZ-diabetic ApoE^−/−^ mice, and STZ-diabetic ApoE^−/−^ mice treated with acacetin (Figure 8). As reported previously, the signaling molecules SOD1, SOD2, Sirt1, PGC-1α, and Sirt3 were remarkably reduced (Figures 8A,B), pAMPK was decreased (Figure 8D), the anti-apoptotic protein Bcl-2 was downregulated, and the pro-apoptotic Bax was increased (Figures 8A–C) in STZ-diabetic ApoE^−/−^ mice (*n* = 8, *p* = 0.0058–0.00011 vs. control). The alterations in the expression of these molecules were reversed in STZ-diabetic ApoE^−/−^ mice treated with acacetin (Figures 8A–D) (*p* = 0.0399–0.0031 vs. STZ-diabetes). The immunostainings of aortic root sections, like in HUVECs cultured with high glucose, showed a decreased expression of Sirt3 in STZ-diabetic ApoE^−/−^ mice, which were upregulated by acacetin treatment (Figure 8E). These results suggest that the natural flavone acacetin confers vascular protection against diabetic atherosclerosis by reversing the downregulation of SOD1, SOD2, PGC-1α, Sirt1, Sirt3, pAMPK, and ratio of Bcl-2/Bax.

## Discussion

In the present study, we demonstrated in HUVECs that acacetin is strongly protective against high glucose insult-induced vascular endothelial injury and involves Sirt3-mediated amelioration of mitochondrial oxidative stress and reduction in mitochondrial dysfunction. The vascular protection of acacetin was confirmed in diabetes-accelerated atherosclerosis in STZ-diabetic ApoE^−/−^ mice with an attenuated vascular lesion progression without affecting blood glucose. This study is the first to demonstrate the anti-atherosclerotic effect of acacetin and its potential mechanisms against hyperglycemic vascular endothelial cells injury, suggesting that acacetin may be a potential therapeutic drug candidate for reducing cardiovascular complications in diabetic patients.

Our previous studies have reported that acacetin is effective in treating atrial fibrillation by selectively blocking atrial potassium currents (I_Kur_, I_KACh_, and sK_Ca_) (Li et al., 2008; Wu et al., 2011; Liu et al., 2016a; Chen et al., 2017), and is cardioprotective against ischemia/reperfusion injury via anti-oxidation and inhibiting apoptosis and inflammation by activating AMPK/Nrf2 signaling (Liu et al., 2016b; Wu et al., 2018). It also has anticancer (Fong et al., 2010; Kim et al., 2014), anti-peroxidation properties, provides neuroprotection against inflammation (Yin et al., 2008; Bu et al., 2019), and reduces E-selectin expression in endothelial cells by regulating MAP kinase (Tanigawa et al., 2013). The present study demonstrates the novel information that acacetin provides strong protection against high glucose-induced vascular endothelial injury and attenuates diabetes-accelerated atherosclerosis in STZ-diabetic ApoE^−/−^ mice.

It is generally recognized that atherosclerosis is a multifactorial vascular progressive disorder involving alteration of several cellular and molecular events in diabetes (Gregg et al., 2016; Madonna et al., 2018). These include the impairment of mitochondrial morphology and function associated with downregulation of SOD, pAMPK and PCG-1α, etc., leading to decrease in ATP production and increase in ROS production (Gregg et al., 2016; Madonna et al., 2018). In the present study, diabetic atherosclerotic lesions were associated with downregulation of signaling molecules (i.e. SOD, Bcl-2, PGC-1α, pAMPK, Sirt3 and Sirt1) in artery tissues in STZ-diabetic ApoE^−/−^ mice and also in HUVECs cultured with high glucose medium.

There are seven human sirtuin isoforms (Sirt1-Sirt7) located in nucleus (Sirt1, Sirt6, and Sirt7), mitochondria (Sirt3, Sirt4, and Sirt5), or cytoplasm (Sirt2) that are activated by NAD^+^ and NAMPT (Imai and Guarente, 2016; Wang et al., 2019). Sirt1 is widely studied for its involvement in nutrient (energy) sensing and various adaptive pathways. Sirt3 which is located mainly in mitochondria regulates metabolism and oxidative stress (Gallardo-Montejano et al., 2016; Yu et al., 2017). The present study demonstrated that mitochondrial Sirt3 expression was reduced concurrently with decreases in Sirt1, pAMPK and PGC-1α in artery tissues from STZ-diabetic ApoE^−/−^ mice and also in cultured HUVECs with high glucose-induced injury. In this study, we reported that Sirt3 is also involved in protecting against diabetic atherosclerosis. It appears that Sirt3 plays a crucial role in acacetin-mediated regulation of mitochondrial function including oxidation balance and ATP generation. Silencing Sirt3 abolishes the protective effect of acacetin against high glucose-induced ROS production and apoptosis, and ATP reduction in HUVECs, but did not affect acacetin-induced upregulation of Sirt1, pAMPK, and PGC-1α. This suggests that Sirt3 is not involved in regulation of Sirt1, pAMPK or PGC-1α.

However, silencing Sirt1 significantly decreased expression of Sirt3, pAMPK and PGC-1α and completely abolishes acacetin-induced upregulation of these molecules in HUVECs cultured with high glucose medium, whereas the AMPK inhibitor dorsomorphin reduced expressions of Sirt3, pAMPK and PGC-1α and fully inhibited acacetin-induced upregulation of pAMPK and PGC-1α (and partially inhibited Sirt3 upregulation by acacetin). AMPK inhibition does not affect Sirt1 expression or acacetin-induced upregulation. These results indicate that protection of acacetin against vascular hyperglycemic injury is related to Sirt1-mediated activation of Sirt3 and AMPK, followed by AMPK-dependent PGC-1α activation which regulates biosynthesis (Cantó et al., 2009; Joseph et al., 2012) and ATP production (Ke et al., 2018), thereby slowing down atherosclerotic progression via inhibiting oxidation and apoptosis in STZ-diabetic ApoE^−/−^ mice.

The clinical drug metformin (Wang et al., 2017) and a number of natural bioactive compounds (Moss et al., 2018; Lin et al., 2020) have been reported to have anti-atherosclerotic effect, including curcumin, quercetin, puerarin, resveratrol, etc. by activating Sirt1 and/or AMPK. However, most of these promising natural compounds face the problem of poor bioavailability. The present study showed that acacetin, in addition to activating pAMPK as previously reported (Wu et al., 2018), increases Sirt1, Sirt3, and PGC-1α activity and is therefore a novel activator of Sirt1 at lower concentration range (0.3–3 μM) than other previously reported activators (>10 μM) with similar activation mechanism, i.e. increasing NAD^+^/NADH ratio. Furthermore, the water-solubility of acacetin is low (0.023 μM) and therefore oral bioavailability is poor. The issues of water-solubility and bioavailability of acacetin have been solved by synthesis of water-soluble prodrug for injection administration, which can be used clinically in the future to not only treat atrial fibrillation, myocardial ischemia/reperfusion injury, but also diabetic atherosclerosis.

There are several limitations in the present study. It was previously reported that acacetin inhibited adipogenesis in adipocytes and in obese mice (Liou et al., 2017). In the present study, we observed that acacetin reversed the alterations of lipid profiles in STZ-diabetic ApoE^−/−^ mice; decreasing the elevated triglyceride, total cholesterol, low-density lipoprotein, lipoprotein A, and lipoprotein B and increasing the reduced high-density lipoprotein. The effects of acacetin on the lipid profile might contribute to the reduction of atherosclerotic process. One of limitations was that characterization (mass, lipid content etc.) of liver and adipose tissues and the related mechanisms of lipid regulation by acacetin were not explored in detail, which remain to be clarified in the future. Second limitation was that Sirt1 and Sirt3 activities in response to acacetin was not evaluated to demonstrate how NAD^+^ increases Sirt1 and/or Sirt3 activity by changing the deacetylase capacity. Third limitation was that HUVECs were used as a cell model in the present study, which may not be a perfect cell model for correlating atherosclerosis. Fourth limitation was that we did not use D-mannitol to serve as osmotic control in HUVECs, since no effect of D-mannitol (27.5 mM with 5.5 mM glucose) on cell viability was observed in the preliminary experiment, which is similar to the observation from the recent report (Niu et al., 2019). However, these limitations will not affect the conclusion that acacetin is effective in protection against vascular endothelial injury induced by high glucose.

Collectively, this study demonstrates the novel pharmacological effect that acacetin is effective in protecting against vascular endothelial cells injury induced by hyperglycemia by preserving mitochondrial function via Sirt1-mediated activation of Sirt3/AMPK/PGC-1α signaling molecules, thereby attenuating diabetes-accelerated atherosclerosis. These results indicate that acacetin may be a promising therapeutic drug candidate for slowing the development of atherosclerosis in patients with diabetes.

## Data Availability Statement

The raw data supporting the conclusions of this article will be made available by the authors, without undue reservation, to any qualified researcher.

## Ethics Statement

The animal study was reviewed and approved by Animal Care and Ethics Committee of Xiamen University, Ximen, China.

## Author Contributions

W-MH, G-RL and YW designed the study. W-MH and X-CC acquired the experimental data. YW provided the financial support. W-MH and G-RL analyzed the data. W-MH draft the manuscript, G-RL revised the article critically for important intellectual content. All authors have read and approved the final manuscript.

## Funding

This study was supported by a Science and Technology Cooperation Fund (U1605226) across the Taiwan Straits of the National Natural Science Foundation of China.

## Conflict of Interest

G-RL was employed in part by Nanjing Amazigh Pharma Limited, Nanjing, China.

The remaining authors declare that the research was conducted in the absence of any commercial or financial relationships that could be construed as a potential conflict of interest.

## Abbreviations

DCFH-DA, 2′,7′-dichlorofluorescin diacetate; DMSO, dimethyl sulfoxide; HE, hematoxylin and eosin; HVUVECs, human umbilical vein endothelial cells; MDA, malondialdehyde; NAD+, oxidized nicotinamide adenine dinucleotide; MTT, 3-(4,5-dimethyl-2-thiazolyl)-2,5-diphenyl-2-H-tetrazolium bromide, NADH, nicotinamide adenine dinucleotide hydride (reduced form of nicotinamide adenine dinucleotide); NAMPT, nicotinamide phosphoribosyltransferase; OCT, optimal cutting temperature; PBS, phosphate buffered saline; ROS, reactive oxygen species; siRNA, small interfering RNA; SDS, sodium dodecyl sulfate; STZ, streptozotocin.
