# Supplementary material for: Acacetin Protects Against High Glucose-Induced Endothelial Cells Injury by Preserving Mitochondrial Function via Activating Sirt1/Sirt3/AMPK Signals
Source: Front Pharmacol. 2020 Dec 18;11:607796. doi: 10.3389/fphar.2020.607796 (PMC7844858; doi:10.3389/fphar.2020.607796)
Supplement: Supplementary file 1 [file datasheet1.pdf]

 DOI: 10.3389/fphar.2020.607796

Supplementary Materials

Table S1, S2 and Figure S1

Table S1. Antibody information

Antibodies	Dilutions	Suppliers	Cat. number
Anti-Sirt1	1:1000	Abcam (Cambridge, MA, USA)	ab32441
Anti-AMPK	1:1000	Cell Signaling (Danvers, MA, USA)	#2532
Anti-pAMPK	1:1000	Cell Signaling (Danvers, MA, USA)	#2535
Anti-SOD1	1:1000	Abcam (Cambridge, MA, USA)	ab183881
Anti-CD31	1:1000	Abcam	ab9498
Anti-Bcl-2	1:1000	Abcam	ab182858
Anti-Bax	1:1000	Abcam	ab32503
Anti-SOD2	1:1000	Abcam	ab68155
Anti-Sirt3	1:1000	Abcam	ab246522
Anti-Sirt3	1:200	Solarbio Technology (Beijing, China)	K005158P
Anti-PGC-1α	1:1000	Abcam	ab191838
Anti-VDAC1	1:1000	Abcam	ab154856
Anti-β-actin	1:1000	Santa Cruz (Dallas, TX, USA)	sc-47778
Peroxidase AffiniPure Goat Anti-Rabbit IgG（H+L）	1:10000	Jackson (West Grove, PA, USA)	111-035-003
Peroxidase AffiniPure Goat Anti-Mouse IgG（H+L）	1:10000	Jackson (West Grove, PA, USA)	115-035-003
Donkey anti-Rabbit IgG (H+L) Highly Cross-Adsorbed Secondary Antibody, Alexa Fluor 488	1:500	Thermo Fisher Scientific (Waltham, MA, USA)	A-21206
Donkey anti-Mouse IgG (H+L) Highly Cross-Adsorbed Secondary Antibody, Alexa Fluor 594	1:500	Thermo Fisher Scientific (Waltham, MA, USA)	A-21203

 DOI: 10.3389/fphar.2020.607796

Table S2. Changes in bodyweight and lipid profiles in ApoE-/- mice

Group	Control (n=8)	Acacetin (n=8)	STZ (n=8)	STZ + acacetin (n=8)
Initial body weight (g)	25.13±1.36	25.01±1.47	24.44±1.68	24.69±1.81
Final body weight (g)	31.22±1.77	29.35±1.82	25.91±1.24	27.87±1.47
Weight change (g)	6.09±1.56	4.34±1.65	1.47±1.46	3.18±1.64
FBG ($mmol \cdot L^{-1}$)	6.2±1.8	7.5±1.6	18.4±1.8**	17.8±1.7**
RBG ($mmol \cdot L^{-1}$)	7.5±1.4	9.1±1.8	23.1±2.4**	20.4±2.1**
TG ($mmol \cdot L^{-1}$)	1.95±0.13	2.18±0.15	3.99±0.39**	2.69±0.2$^{\#\#}$
TC ($mmol \cdot L^{-1}$)	14.22±0.82	14.98±1.02	21.72±1.13**	16.29±0.95$^{\#}$
HDL ($mmol \cdot L^{-1}$)	0.73±0.05	0.78±0.08	0.49±0.03**	0.66±0.04$^{\#}$
LDL ($mmol \cdot L^{-1}$)	0.9±0.03	1.0±0.03	6.39±0.49**	4.39±0.53$^{**,\#\#}$
LP(A) ($mmol \cdot L^{-1}$)	14.10±1.40	18.25±2.10	70.33±9.60**	27.93±4.60$^{\#\#}$
LP(B) ($mmol \cdot L^{-1}$)	0.075±0.011	0.076±0.010	0.154±0.013**	0.078±0.013$^{\#\#}$

FBG, fasting blood glucose; RBG, random blood glucose； TG，triglyceride; TC, total cholesterol; LDL, low-density lipoprotein; HDL, high-density lipoprotein; LP (A), lipoprotein A; LP (B), lipoprotein B. $^{*}P<0.05$, $^{**}P<0.01$ vs. control; $^{\#}P<0.05$, $^{\#\#}P<0.01$ vs. STZ.

Supplementary Figure S1

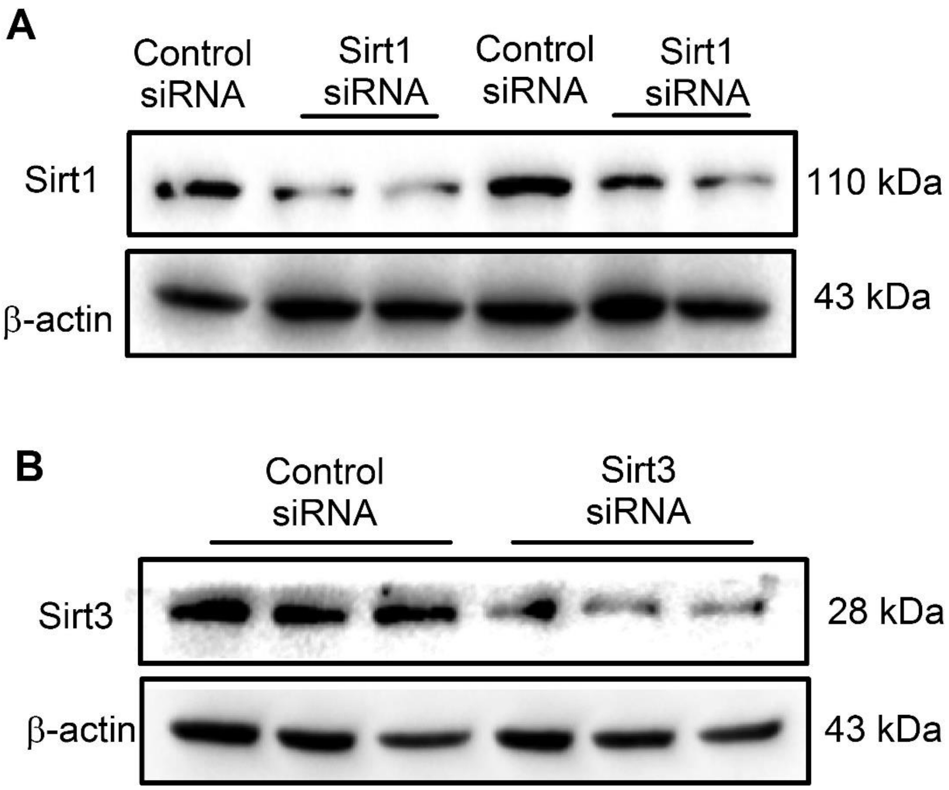

Figure S1. Silencing efficiency of Sirt1 (A) and Sirt3 (B) in human umbilical vein endothelial cells (HUVECs). The cells were transfected with 60 nM control siRNA, Sirt1 siRNA, or Sirt3 siRNA using Lipofectamine RNAiMAX Reagent following the manufacturer's instruction for 48 h, and then exposed to 33 mM glucose for 5 days.
