# Supplementary material for: Acacetin Protects Against High Glucose-Induced Endothelial Cells Injury by Preserving Mitochondrial Function via Activating Sirt1/Sirt3/AMPK Signals
Source: Front Pharmacol. 2020 Dec 18;11:607796. doi: 10.3389/fphar.2020.607796 (PMC7844858; doi:10.3389/fphar.2020.607796)

Acacetin protects against high glucose-induced endothelial cells injury by preserv-ing mitochondrial function via activating Sirt1/Sirt3/AMPK signals

Wei-Min Han, Xu-Chang Chen, Yan Wang, Gui-Rong Li

Original Western blot images showed in the manuscript figures

Figure 1E

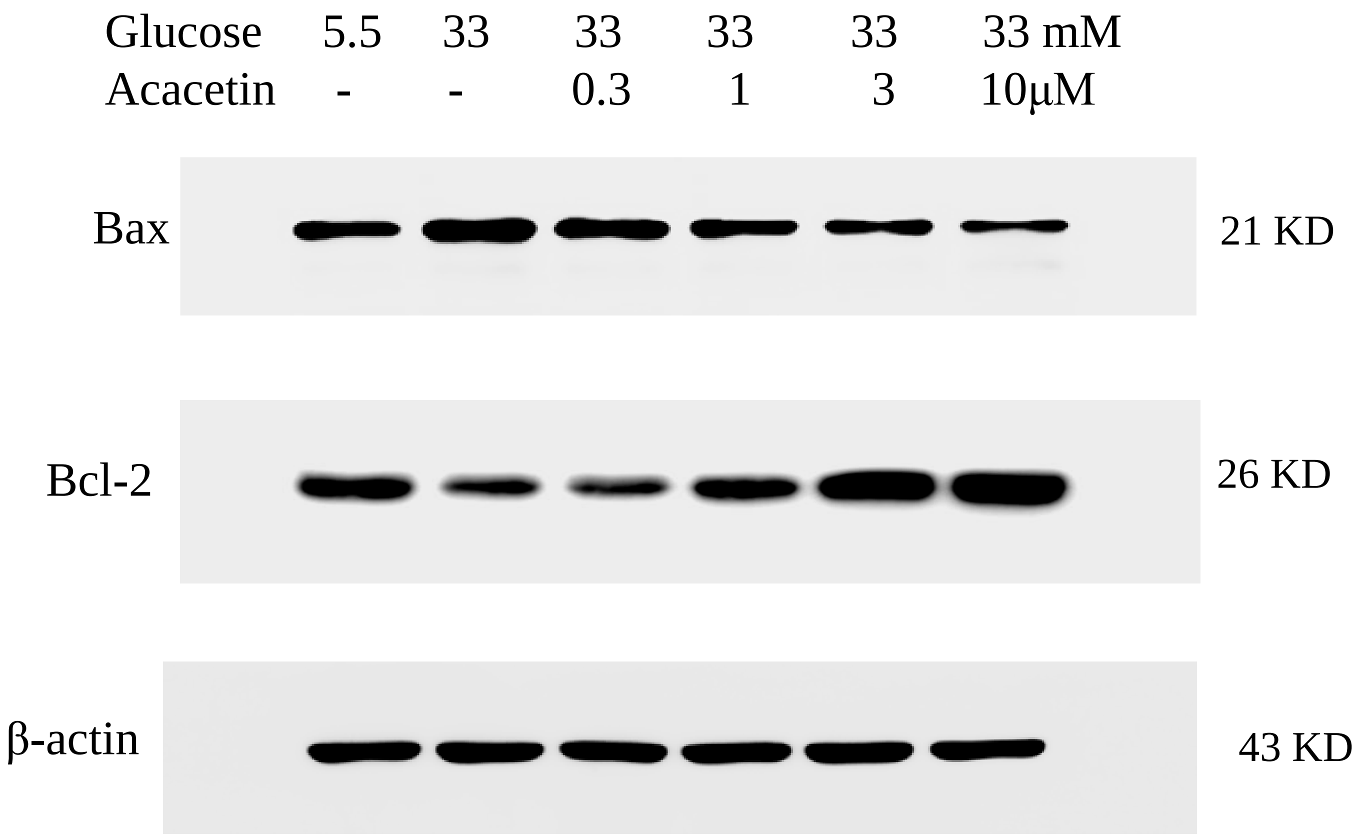

Glucose 5.5 33 33 33 33 33 mM
Acacetin - - 0.3 1 3 10μM
Bax
21 KD
Bcl-2
26 KD
β-actin
43 KD

Figure 2E

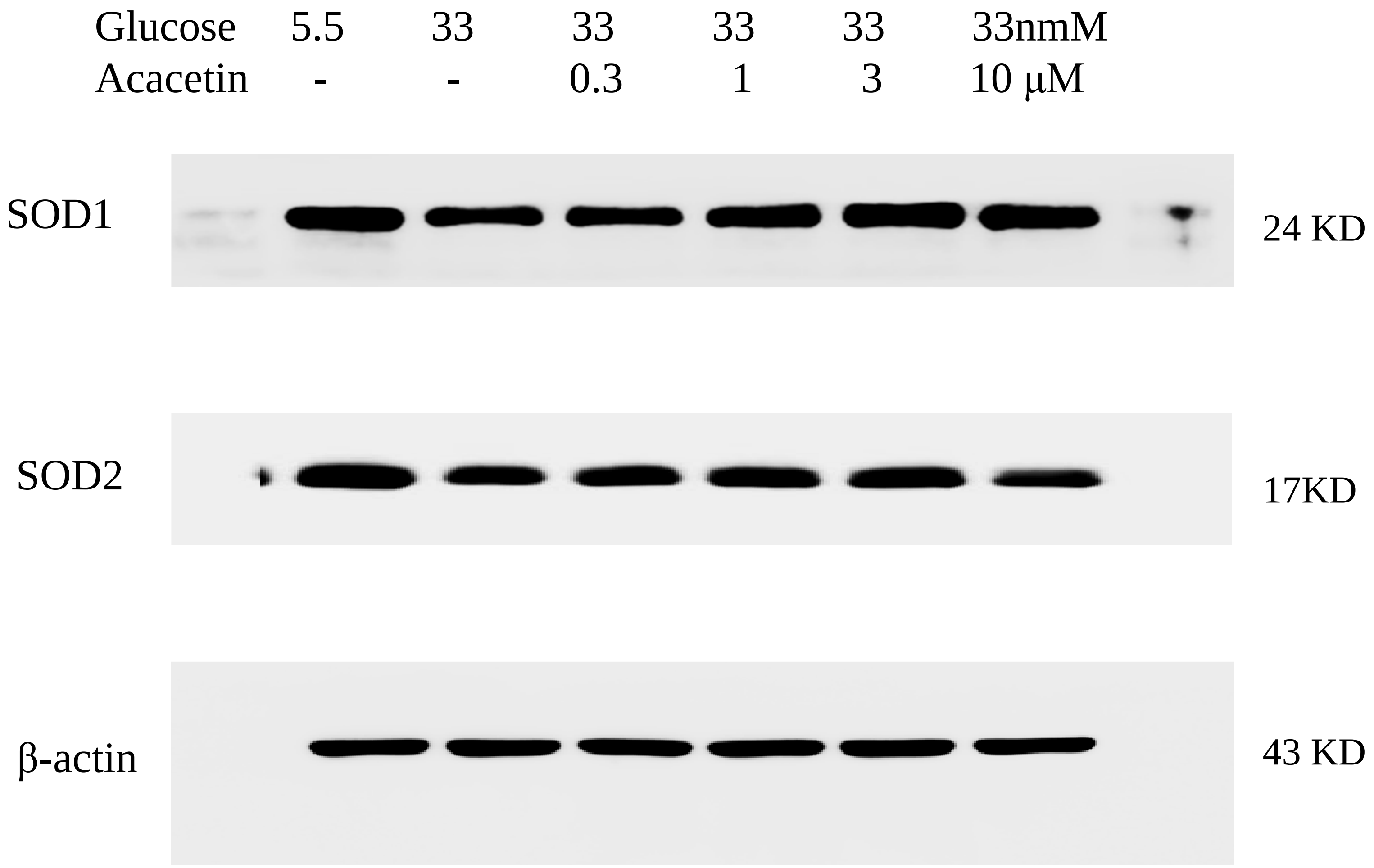

Glucose 5.5 33 33 33 33 33nmM
Acacetin - - 0.3 1 3 10 μM
SOD1
24 KD
SOD2
17KD
β-actin
43 KD

Figure 3E

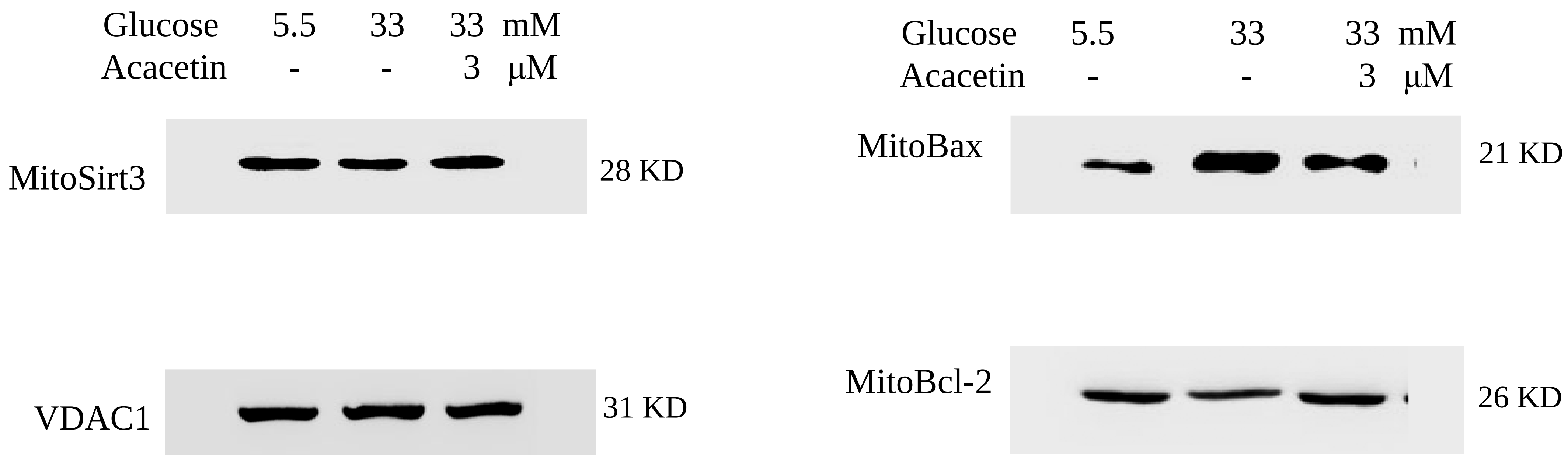

Glucose 5.5 33 33 mM
Acacetin - - 3 μM
MitoSirt3
28 KD
VDAC1
31 KD
Glucose 5.5 33 33 mM
Acacetin - - 3 μM
MitoBax
21 KD
MitoBcl-2
26 KD

Figure 5A

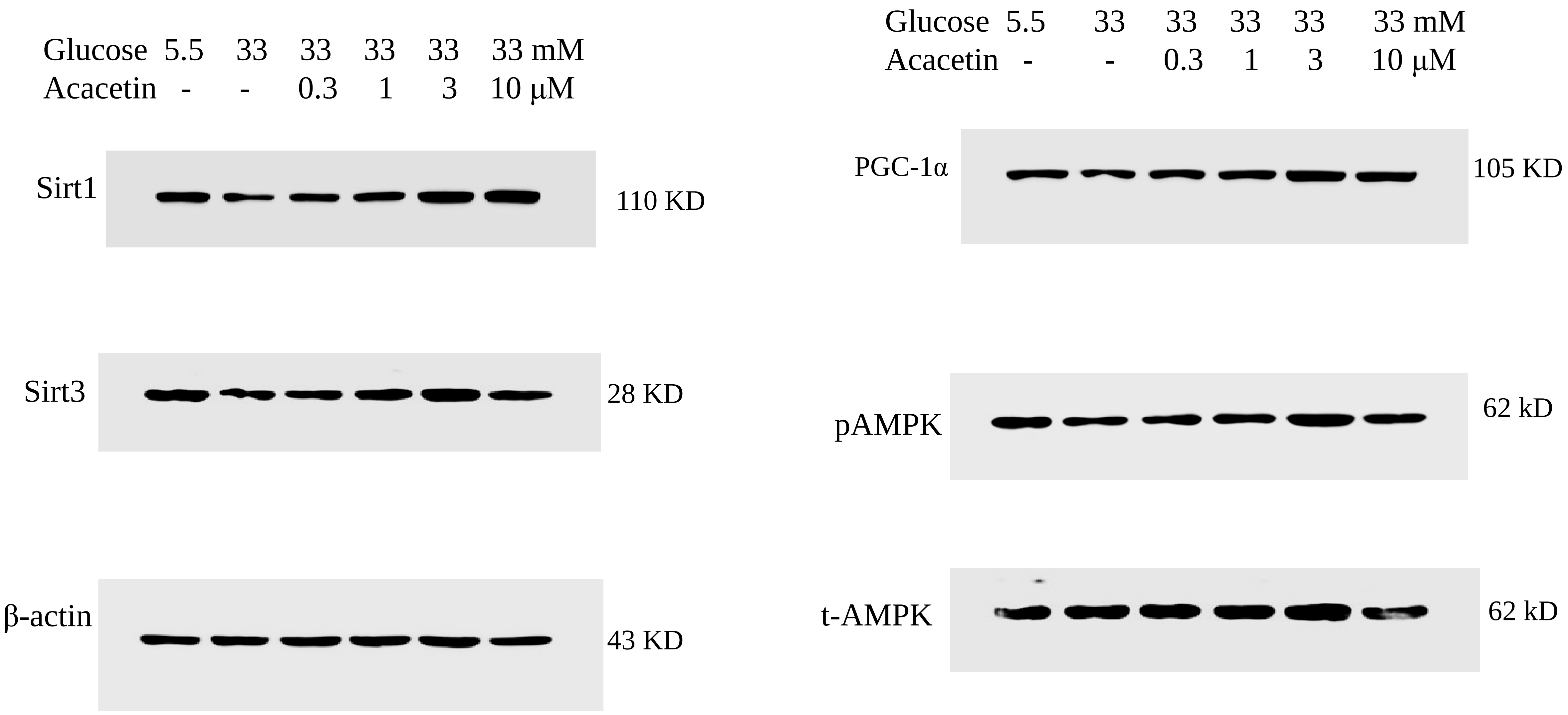

Glucose 5.5 33 33 33 33 33 mM
Acacetin - - 0.3 1 3 10 μM
Sirt1
110 KD
Sirt3
28 KD
β-actin
43 KD
Glucose 5.5 33 33 33 33 33 mM
Acacetin - - 0.3 1 3 10 μM
PGC-1α
105 KD
pAMPK
62 kD
t-AMPK
62 kD

Figure 6A

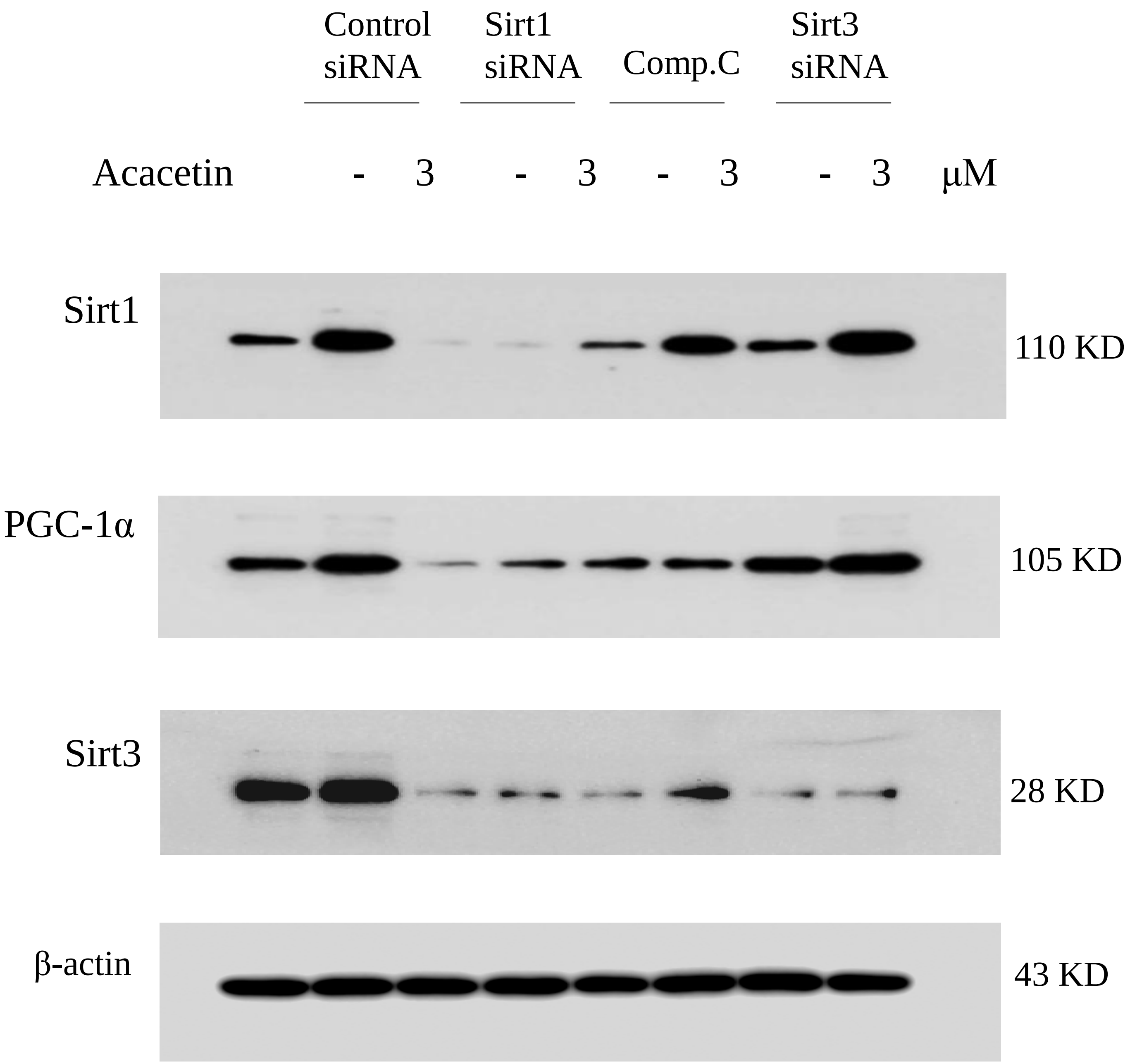
Control siRNA
Sirt1 siRNA
Comp.C
Sirt3 siRNA
Acacetin - 3 - 3 - 3 - 3 μM
Sirt1
110 KD
PGC-1α
105 KD
Sirt3
28 KD
β-actin
43 KD

Figure 6A

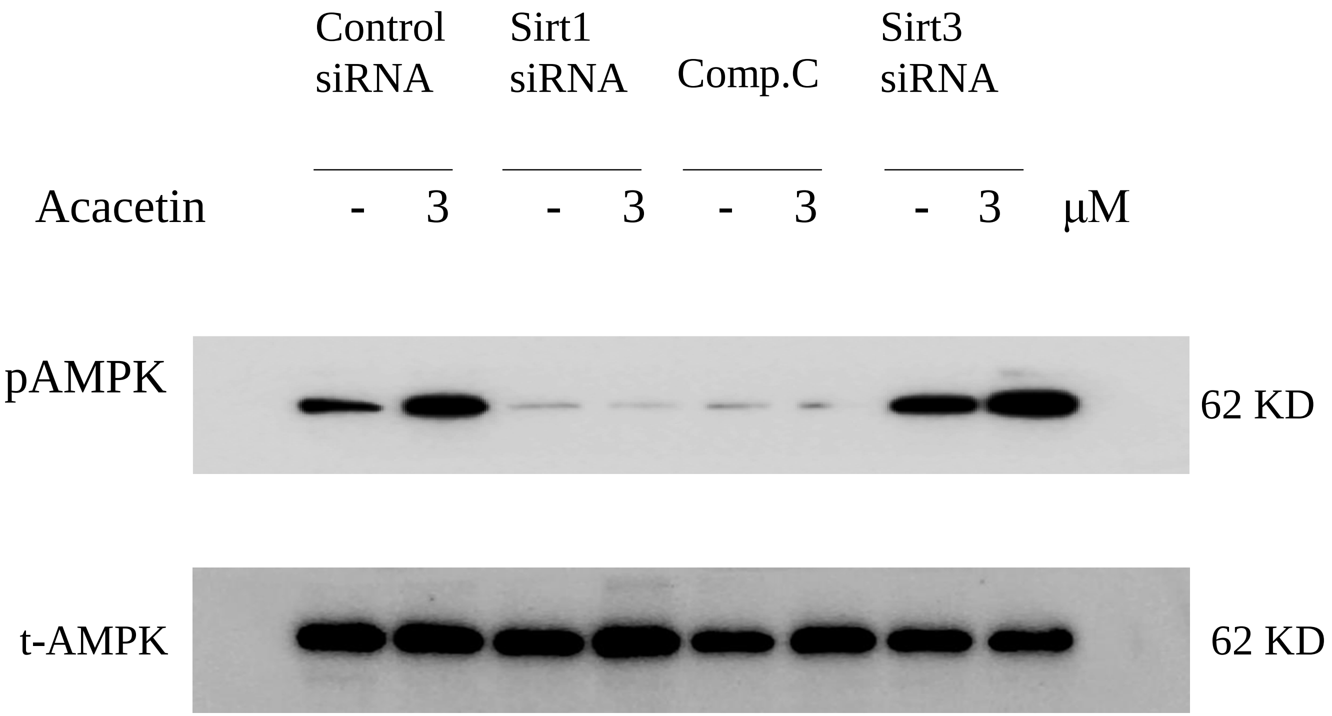

Control siRNA
Sirt1 siRNA
Comp.C
Sirt3 siRNA
Acacetin
- 3 - 3 - 3 - 3 μM
pAMPK
62 KD
t-AMPK
62 KD

Figure 6D

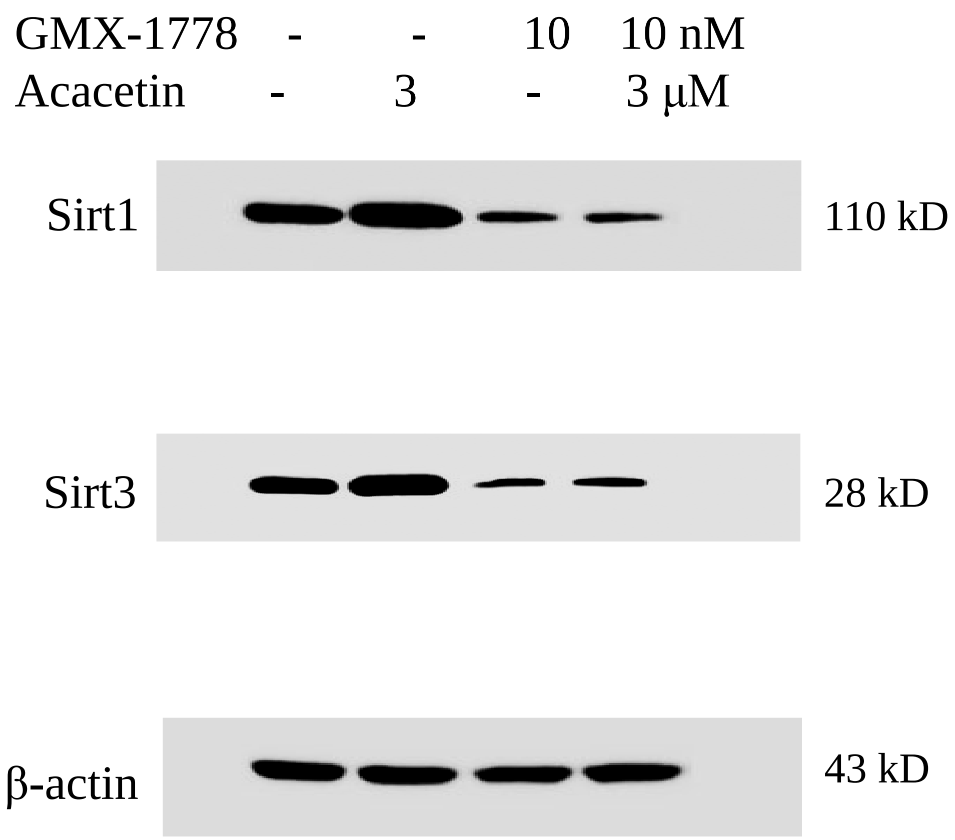
GMX-1778 - - 10 10 nM
Acacetin - 3 - 3 μM
Sirt1
110 kD
Sirt3
28 kD
β-actin
43 kD

Figure 8A

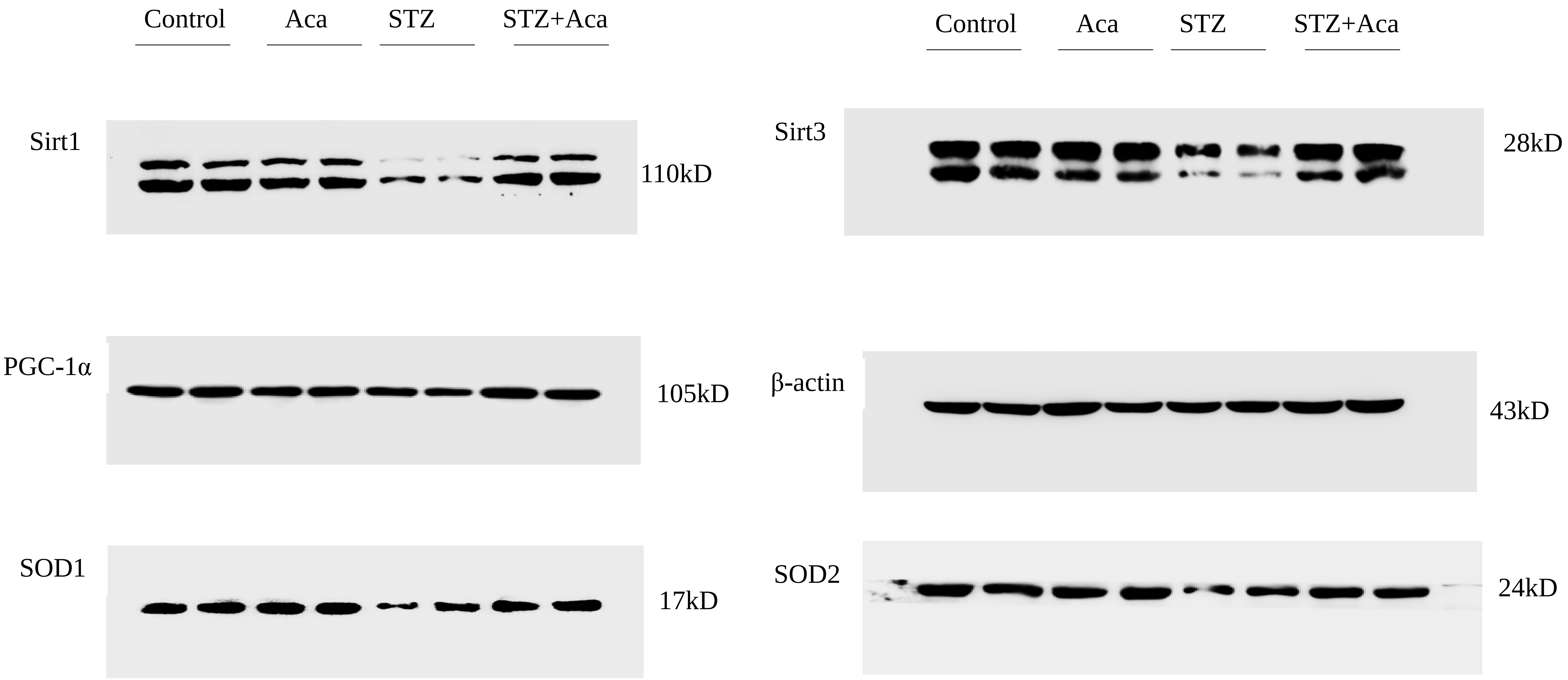

Control
Aca
STZ
STZ+Aca
Sirt1
110kD
PGC-1α
105kD
SOD1
17kD
Control
Aca
STZ
STZ+Aca
Sirt3
28kD
β-actin
43kD
SOD2
24kD

Figure 8A

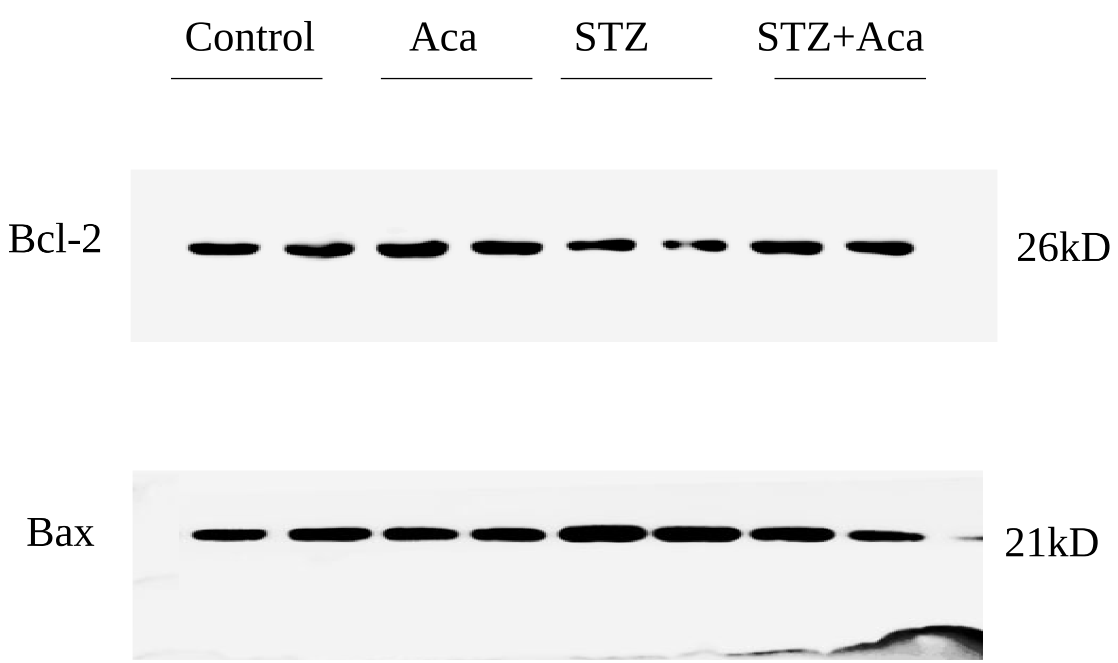

Figure 8D

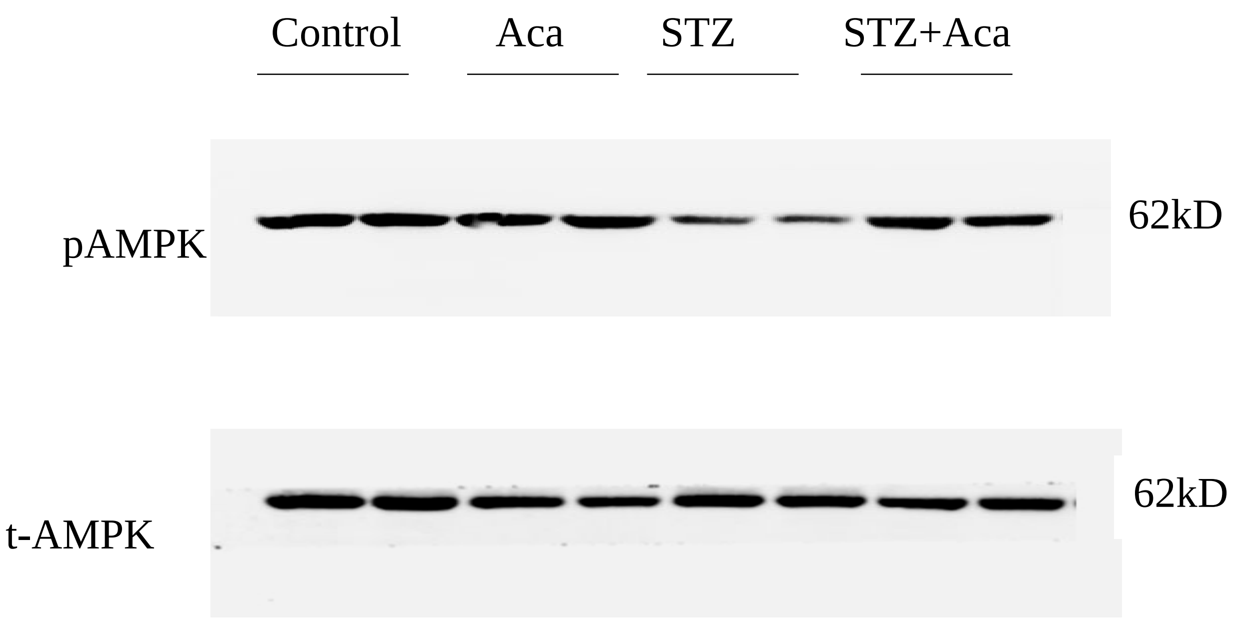

Supplement: Supplementary file 2 [file datasheet2.pdf]
